# Chemometric Strategies for Fully Automated Interpretive Method Development in Liquid Chromatography

**DOI:** 10.1021/acs.analchem.2c03160

**Published:** 2022-11-01

**Authors:** Tijmen S. Bos, Jim Boelrijk, Stef R. A. Molenaar, Brian van ’t Veer, Leon E. Niezen, Denice van Herwerden, Saer Samanipour, Dwight R. Stoll, Patrick Forré, Bernd Ensing, Govert W. Somsen, Bob W. J. Pirok

**Affiliations:** † Division of Bioanalytical Chemistry, Amsterdam Institute of Molecular and Life Sciences, Vrije Universiteit Amsterdam, De Boelelaan 1085, 1081HVAmsterdam, The Netherlands; ‡ AMLab, Informatics Institute, University of Amsterdam, Science Park 904, 1098 XHAmsterdam, The Netherlands; § Analytical Chemistry Group, Van’t Hoff Institute for Molecular Sciences, University of Amsterdam, Science Park 904, 1098XHAmsterdam, The Netherlands; ∥ Centre for Analytical Sciences Amsterdam (CASA), Science Park 904, 1098XHAmsterdam, The Netherlands; ⊥ AI4Science Lab, University of Amsterdam, Science Park 904, 1098XHAmsterdam, The Netherlands; # Computational Chemistry Group, Van’t Hoff Institute for Molecular Sciences, University of Amsterdam, Science Park 904, 1098XHAmsterdam, The Netherlands; ¶ Department of Chemistry, Gustavus Adolphus College, Saint Peter, 56082Minnesota, United States

## Abstract

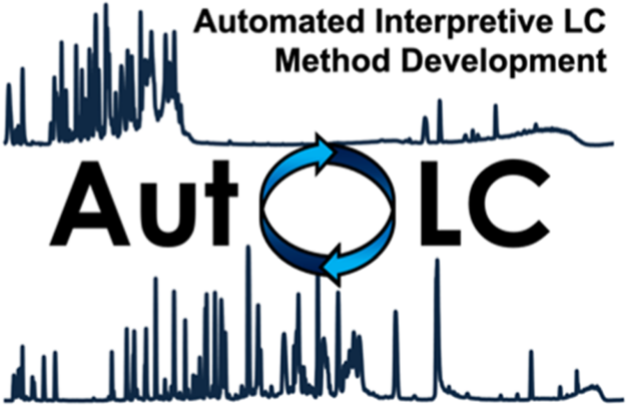

The majority of liquid chromatography (LC) methods are still developed in a conventional manner, that is, by analysts who rely on their knowledge and experience to make method development decisions. In this work, a novel, open-source algorithm was developed for automated and interpretive method development of LC(−mass spectrometry) separations (“AutoLC”). A closed-loop workflow was constructed that interacted directly with the LC system and ran unsupervised in an automated fashion. To achieve this, several challenges related to peak tracking, retention modeling, the automated design of candidate gradient profiles, and the simulation of chromatograms were investigated. The algorithm was tested using two newly designed method development strategies. The first utilized retention modeling, whereas the second used a Bayesian-optimization machine learning approach. In both cases, the algorithm could arrive within 4–10 iterations (*i.e.*, sets of method parameters) at an optimum of the objective function, which included resolution and analysis time as measures of performance. Retention modeling was found to be more efficient while depending on peak tracking, whereas Bayesian optimization was more flexible but limited in scalability. We have deliberately designed the algorithm to be modular to facilitate compatibility with previous and future work (*e.g.*, previously published data handling algorithms).

## Introduction

A major component of method development in liquid chromatography (LC) is the selection of kinetic (*e.g.*, column length and particle size) and thermodynamic (*e.g.*, gradient profiles and temperature) parameters. To tackle this problem, several tools utilizing design-of-experiment workflows and retention modeling based on experimental data and/or chemical structure information have been developed and even commercialized. Notable examples of the latter include DryLab^1^ (Molnar Institute), ChromSword^2^ (Merck KGaA), and LC & GC Simulator^3,4^ (ACD/Labs). Meanwhile, researchers all over the world continued to improve method development workflows, by further developing retention models^5^ and kinetic plots for kinetic parameter selection,^6^ accounting for injection profiles,^7^ evaluating alternative retention mechanisms such as HILIC,^8,9^ and investigating the use of neural networks for retention modeling and method optimization.^10^

The prospect of simplifying method development is particularly vital for two-dimensional LC (2D-LC) which still is challenging to use routinely, requiring a large number of parameters to be simultaneously optimized relative to 1D-LC.^11,12^ Innovations in modulation technology have improved the compatibility and sensitivity of the technique^13,14^ but arguably rendered numerical optimization of all parameters even more daunting. To support innovation in research and society, this challenge must be addressed.

Our groups have previously proposed a workflow for the optimization of gradient parameters in comprehensive 2D-LC,^15^ inspired by the work of Dolan and co-workers^1^ and Schoenmakers.^16^ Schoenmakers was inspired by the interpretive design of the work by Laub and Purnell for GC.^17^ The term interpretive refers to the capability of the workflow to improve a method by unsupervised interpretation of chromatographic data (*i.e.* data from a scouting method). The utility of this approach is that the workflow can be applied to samples of unknown composition. This is in stark contrast to most of the previously listed approaches in which the user is required to specify chemical structural information or retention times of all compounds of interest. The latter requires that sample complexity is limited and a mass spectrometer is available. This prospect is not feasible for the highly complex mixtures usually targeted by ultrahigh-performance LC (UHPLC) and LC × LC methods. Indeed, although our previous workflow for developing 2D-LC methods was a step in the right direction, a fair criticism of that work was that manual assignment of all peaks was not practically feasible.

Fortunately, the chemometrics community has developed a plethora of tools that may support method development, including but not limited to peak detection, background correction, peak tracking optimization algorithms, and optimization criteria.^18^ However, these tools often require a high level of expertise to be used effectively. To make things worse, there are very few published studies that critically compare and numerically evaluate the developed algorithms.^18^ For example, we found 15 background correction algorithms developed in the past 10 years, in addition to the plethora of existing metrics for background correction, yet not a single study that offered any meaningful comparison of their performance.^19^ Nevertheless, these and many other developments, including the use of artificial intelligence,^20^ look promising as tools that could potentially accelerate method development.

The number of parameters that can be adjusted to fine-tune LC and mass spectrometry (MS) methods is too high to routinely implement their optimization during method development. Thus, many users resort to trial-and-error and experience-driven selection of method parameters. It would thus be advantageous to combine the best available theory and tools from the chromatographic and chemometric communities into automated closed-loop method development systems. Such strategies are not new. Indeed, I and co-workers investigated the use of decision trees for LC optimization for four pharmaceutical compounds.^21^ The group of Kell published their robot chromatographer system for gas chromatography (GC)–MS in metabolomics^22,23^ and later extended it for a one-step optimization for LC–MS.^24^ Their solution utilized a PESA-II genetic multiobjective optimization algorithm operated using a combination of Microsoft Excel and mouse-click macros. For LC, Susanto *et al.* also applied a multiobjective genetic algorithm to find optimal separation conditions for the three proteins, lysozyme, ribonuclease A, and cytochrome *C* in gradient LC.^25^ More recently, Bradbury *et al.* introduced the MUSCLE software^26,27^ to develop an LC–MS/MS method for several vitamin D metabolites. While impressive, the above works focus exclusively on very specific applications that typically involve a limited number of analytes; thus, these approaches are difficult to generalize.

To address this challenge, here we present an interpretive algorithm workflow for automated LC–MS and LC-DAD method development (“AutoLC”) suitable for complex samples. Novel scientific algorithms were developed to facilitate automation including improved LC–MS peak tracking, exhaustive retention modeling, Bayesian optimization (BO), and generation of gradient profiles that potentially yield meaningful improvements. This AutoLC algorithm directly and iteratively programs the LC with new method parameters following the analysis of raw experimental data obtained from previous iterations of the algorithm until convergence of a specified objective function is reached. To our knowledge, this is the first time such an interpretive closed-loop system has been reported for LC. To demonstrate the modularity of the approach, we investigate a strategy based on exhaustive retention modeling and an exploratory strategy based on the machine learning (ML) method called BO. We would like to stress that the workflow was deliberately designed to be modular, to be inclusive, and compatible with other tools published in the literature. Our goal is to publish an open-source tool that all chromatographers can use to their benefit with the ability for others to test and exploit their own algorithms. This tool is the first step of the paradigm shift toward fully automated method development and its prototype is provided in the Supporting Information.

## Experimental Section

### Instrumentation

Two chromatographic systems were used for the experiments.

### System A

System A was an Agilent Infinity II 2D-LC system, with a binary pump (G7120), a Jet Weaver V35 mixer (G7120-68135), an autosampler (G4226A), a column oven (G7116B), and a Q-TOF mass spectrometer (G6549A, MS). A Poroshell HPH-C18 (693675-702, 150 × 2.1 mm, *d*_p_ = 1.9 μm) column was used for all experiments. Control and computations were conducted using a system featuring an AMD Ryzen 9 5950X (16 CPU) on an Asus TUF GAMING X570-PLUS (WI-FI) motherboard. The system featured an NVIDIA Quadro P620 GPU with 4 × 32GB T-FORCE XTREEM ARGB DDR4 running at 3200 MHz.

### System B

System B was an Agilent Infinity II 2D-LC system with a binary pump (G7120), a Jet Weaver V35 mixer (G7120-68135), two 12-pos/13-port bio-inert solvent selection valves (5067-4159), an autosampler (G7129B), column oven (G7116B) outfitted with an 8-pos/18-port valve (5067-4233) for column selection, and a diode-array detector (G7117B, DAD). The system also employed a TraceDec contactless conductivity detector (C4D) connected to the outlet of the mixer to record the actual shape of the gradient. To allow UHPLC conditions, the original probe connection was replaced with a fused-silica capillary. An Agilent 1290 Infinity in-line filter was used in front of the Poroshell 120 SB-C18 (685775-902, 100 × 2.1 mm, *d*_p_ = 2.7 μm) column used for all experiments. Instrument control and AutoLC algorithm computations were all carried out on an AMD Ryzen Threadripper 3970W (32 CPU, 64 Threads) on an Asus ROG STRIX TRX40-XE motherboard with an NVIDIA Quadro RTX 4000 8GB GDDR6 GPU and 8 × 32GB G.Skill DDR4 Ripjaws-V RAM 3200 MHz running at 2666 MHz.

### Chemicals

Milli-Q water (18.2 MΩ cm) was obtained from a purification system (Arium 611UV, Sartorius, Germany). Acetonitrile (LC–MS grade) was obtained from Biosolve (Valkenswaard, The Netherlands). Triethylamine (≥99.5%) and formic acid (reagent grade, ≥95%) were obtained from Sigma-Aldrich (Darmstadt, Germany).

Sample A (retention modeling) was prepared by digesting a monoclonal antibody with trypsin. This is the same sample described and used in two of our prior studies,^28^ one of which found 189 different compounds using MS detection.^29^ Sample B (BO) was a solution of 80 degraded dyestuffs provided by the Dutch Cultural Heritage Agency and was used also in an earlier study.^30^ More details on these mixtures and sample preparation can be found in Supporting Information Section S1.

### Procedures

For system A, a 0.1% formic acid aqueous solution was used as eluent A and acetonitrile as eluent B. The flow rate was set to 0.4 mL•min^–1^. For system B, the mobile phase was a mixture of 95% aqueous 5 mM triethylamine solution brought to pH 3.0 using formic acid and 5% acetonitrile (v/v) (eluent A). The organic modifiers were acetonitrile and 5% aqueous 5 mM triethylamine solution brought to pH 3.0 using formic acid (eluent B). The flow rate was set to 0.65 mL•min^–1^.

### Software

The core AutoLC algorithms were written in Python 3.9 using PyCharm 2021.1.2 (JetBrains, Prague, Czech Republic). The Python environment was set up using Anaconda 3 (Anaconda Inc., Austin, TX, USA). To interface with the LC instrument, an algorithm was written in C++ using Microsoft Visual Studio 2022 (Microsoft, Redmond, WA, USA) to interface with the OpenLAB CDS Chemstation Edition (rev. C.01.10 [287]). For retention modeling, the AutoLC algorithm and signal processing was done in Python 3.9 using PyCharm 2021.1.2 and MATLAB 2021b (Mathworks, Natick, MA, USA), which was used for the peak detection, tracking, and optimization algorithms, whereas peak detection was supported by the findpeaks MATLAB function.^31^ To monitor progress, the AutoLC algorithm was programmed to report its status and data continuously in Slack 4.22 (San Francisco, CA, USA) using the Python Slack SDK.

## Results and Discussion

**Figure 1:**
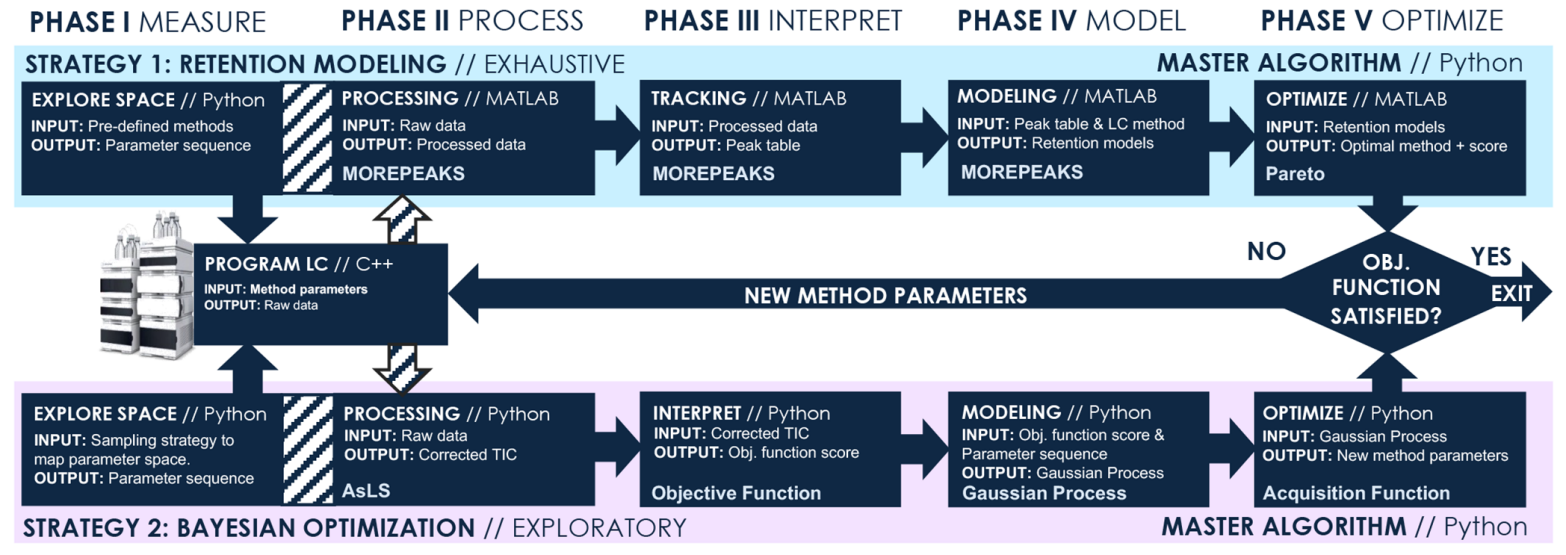
Schematic overview of the generic workflow employed by the AutoLC algorithm using retention modeling (top, blue) or BO (bottom, pink). Optimization through retention modeling with peak tracking.

In anticipation of the inevitable desire to incorporate knowledge beyond the scope of this work (*e.g.*, peak detection strategies published previously, or in the future), the AutoLC algorithm was designed as a chain of independent operations with controlled input and output criteria. In this workflow, which is shown in Figure 1, the LC is used as a subordinate, controlled by the AutoLC algorithm with method parameters and a start signal as input and raw data as output. Further method development iterations (MDI) can be initiated by the AutoLC algorithm without operator intervention until the objective criteria are met.

Although the workflow was inspired by our earlier MOREPEAKS protocol,^15,32^ several scientific challenges had to be addressed with respect to peak tracking, retention modeling, gradient profile optimization, and performance score functions. These will be explained along with the two different optimization strategies that were investigated.

Retention modeling is based on first determining the retention coefficients of analytes through several scouting runs.^33^ These coefficients can then be used to construct retention models that allow the simulation of separations under a large number of different chromatographic conditions (*i.e.*, methods). The separation performance can then be assessed for each simulated method. The method that led to the best-simulated chromatogram can then be selected as optimal and—in our case—directly programmed into the LC system by the AutoLC algorithm without input from the operator. For this workflow, the selection of scouting gradients (Figure 1, phase I) was based on our earlier work,^33^ sampling the modifier fraction (φ)-range with three different gradient slopes. For retention modeling, no data preprocessing (phase II) was conducted.

However, the construction of models for all individual (unknown) analytes requires each to be linked in all measured chromatograms. For this to be conducted automatically, peak tracking algorithms (Figure 1, phase III) can use features related to the peak shape and spectra to search the chromatogram. For this, we developed a new peak tracking algorithm that was based on earlier preliminary work.^34^ In that work, we solely based the peak detection on the total-intensity chromatogram. To improve peak detection in the present work, we added a second peak detection stage which exclusively uses the *m*/*z* data. In brief, the algorithm uses the average *m*/*z* spectrum for the entire chromatogram. Next, the algorithm would iteratively (i) investigate the most intense *m*/*z* signal on the spectrum, (ii) use the extracted ion current signal to investigate whether this *m*/*z* represented a chromatographic peak (one or multiple singular peaks *vs* noise across the chromatogram). In the event that (ii) was true, the algorithm adds *m*/*z* and related retention time to the peak list, and the signal was removed from the full *m*/*z* spectrum. This sequence would reiterate until 80% of the full area of the original full *m*/*z* spectrum was described or no peaks could be found on the current *m*/*z*. This number (80%) was not optimized. Further study is required to investigate the validity of this number.

**Figure 2:**
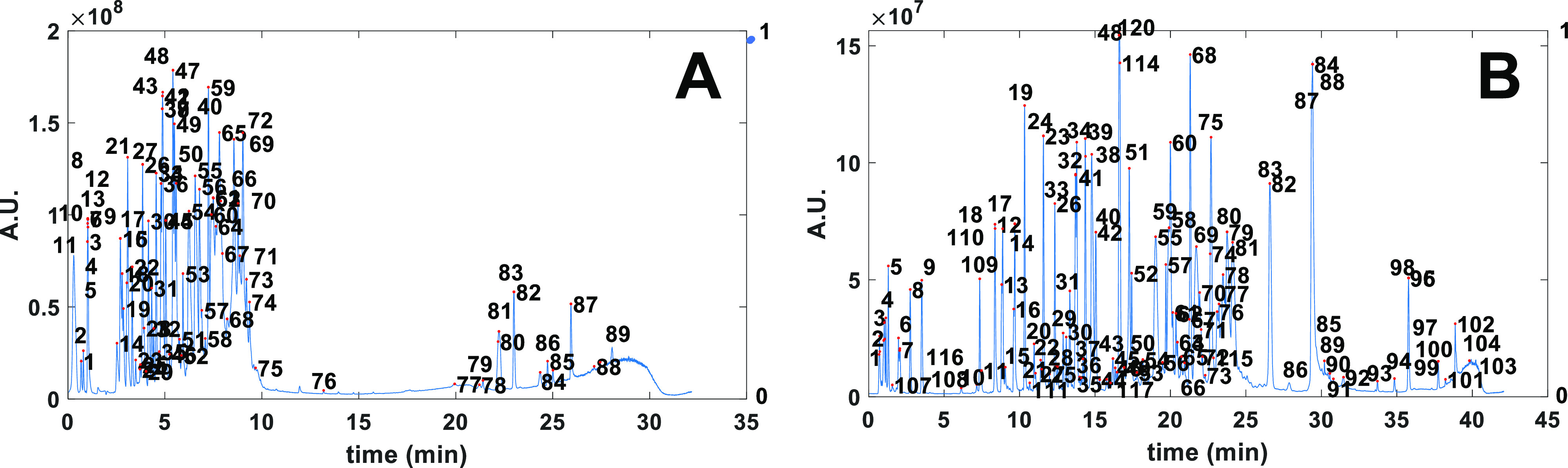
Example of peak tracking results using system A and sample A during automated optimization of LC–MS separations of an antibody digest during MDI 1 (A) and MDI 4 (B). See Supporting Information Section S2 for extended peak tables, and tracked chromatograms for other MDIs. Note that (A) features peak labels as detected and tracked during the first three MDIs, whereas (B) features peak labels after the first retrack (see main text). Consequently, peak numbers do not match in this example. Dotted purple lines depict the programmed solvent gradient (graphically uncorrected for dwell volume).

The results for one of the scouting gradients in MDI 1 and the proposed gradient for MDI 4 are shown in Figure 2A,B, respectively, with the numbers depicting the analytes found and tracked across the two chromatograms. The peak tracking results for all chromatograms, including peak tables and chromatograms can be found in Supporting Information Section S2. One point of concern was that the peak tracking algorithm would exclusively search for analytes found during the scouting runs. However, as the optimization process continued, the likelihood of separating new, previously coeluting compounds increased. Consequently, we developed so-called retrack subroutines after the fourth and ninth MDI (*i.e.*, every 5*n* – 1 MDI), where the algorithm would restart the peak detection process, without using any knowledge from previous separations.

As such a retrack considers all LC–MS chromatograms generated thus far, the computational time needed increases exponentially. Thus, while ideally a retrack would be executed after every single MDI, we opted for every 5 MDI to limit the computational impact. Retracking is only sensible after MDI 4 with data from three scouting gradients and one proposed optimum to consider. The peak tracking table in Supporting Information Section S2 features the composite tracking table after MDI 13. This also explains why more peaks are tracked in MDI 4 (Figure 2B) compared to those tracked in MDI 1 (Figure 2A).

### Retention Modeling

In phase IV, the obtained peak tables were used to create retention models using equations derived for multistep gradients.^35^ For this study, we employed the so-called log-linear exponential (commonly referred to as, LSS) model
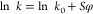
1where *k* is the retention factor, φ is the mobile phase modifier fraction, and *k*_0_ and *S* are fitting coefficients. The LSS model was chosen for this example to minimize the number of scan measurements needed and reduce computation time, recognizing that we sacrifice some accuracy in the model in doing so. For each detected analyte, the algorithm initialized 20 simultaneous fmincon (MATLAB) regressions that each searched for the minimum of a nonlinear multivariable function within set constraints to determine the best fits for *k*_0_ and *S*. Each was allowed to loop for a maximum of 3000 function evaluations using randomized but constrained starting parameters (see Supporting Information Section S3). For each analyte, the best retention model (*i.e.*, lowest sum-of-squared residuals) was then used in phase V for subsequent separation simulations.

### Generation of Meaningful Candidate Gradient Methods

When deciding what method parameters to use in a subsequent MDI, one essential aspect was the generation of candidate gradient methods that would produce meaningful (*i.e.*, better separation and shorter analysis time) improvements over standard linear scouting gradients while also providing flexibility for samples that exhibit multiple peak clusters across a scouting chromatogram. This flexibility was designed into the process by using 16-parameter, 5-segment gradient programs (see Supporting Information Section S4 for a schematic and parameter overview). The five-segment gradient program was chosen to give the algorithm the possibility to form complex gradients providing good separations without making the required computational time unreasonable. Each segment started with an isocratic section of length *t*_*n*_ at φ_*n*_, followed by a gradient section of length *t*_G,*n*_ increasing to φ_*n*+1_. After five such segments, the gradient would end at φ_6_. To avoid situations where one or more analytes would not elute from the column, a final segment was added that immediately set the organic modifier to φ_7_ = 1 and maintain this for a time *t*_final_. For φ, the algorithm was constrained to positive gradient slopes of dφ/d*t* by enforcing φ_*n*_ ≤ φ_*n*+1_. As a time constraint, the analysis was limited to a set *t*_max_, defined as ∑*t*_*n*_ + ∑*t*_G,*n*_ ≤ *t*_max_. In this study, *t*_max_ was limited to 40 min, but this value can be considered case-specific.

However, the increased number of parameters (*i.e.*, gradient segments) needed to provide method flexibility to generic unknown samples also increased the dimensionality of the optimization problem. This rendered the search for candidate methods that actually yield a meaningful improvement with respect to earlier MDIs challenging. Nevertheless, these sophisticated gradients were necessary to allow the algorithm to be generally applicable to samples of unknown composition as is also visually demonstrated by the dotted purple gradient programs plotted in Figure 2 for a linear and segmented gradient. To increase the likelihood of finding useful candidate gradients, a two-stage optimization strategy was investigated. Including the gradient programs from the previous *x* MDIs, the algorithm generated 2000 – *x* new candidate methods with very different parameter values (see Supporting Information Section S4 for further constraints).

### Evaluation of Simulated and Experimental Separations

As a first step of the optimization, each of the 2000 candidate methods was individually optimized using fmincon as described above. For this optimization, the retention times for all analytes were predicted using the equations for multistep gradients as published earlier within the MOREPEAKS environment in MATLAB,^35^ and peak widths were computed using the gradient peak compression model by Hao *et al.*(36) The model by Hao *et al.* required an estimate of the plate number *N*, which was estimated for each peak from the scouting MDI experimental data (*i.e.*, MDI 1–3) by fitting the Hao model to the peak widths for each analyte. For each MDI, the optimization was driven by the evaluation of a resolution score  (lower is better, see Supporting Information Section S5 for a guiding graphical depiction), which was calculated using eq 2
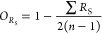
2where ∑*R*_S_ is the adapted sum of all resolution values of all unique neighboring peak pairs within one chromatogram and *n* is the number of peaks detected. Within this sum, each individual *R*_S_ value (*i.e.*, between two adjacent peaks) was set to 2 if the resolution was greater than 2 to penalize unnecessarily large *R*_S_ values and *n* is the number of peaks.

**Figure 3:**
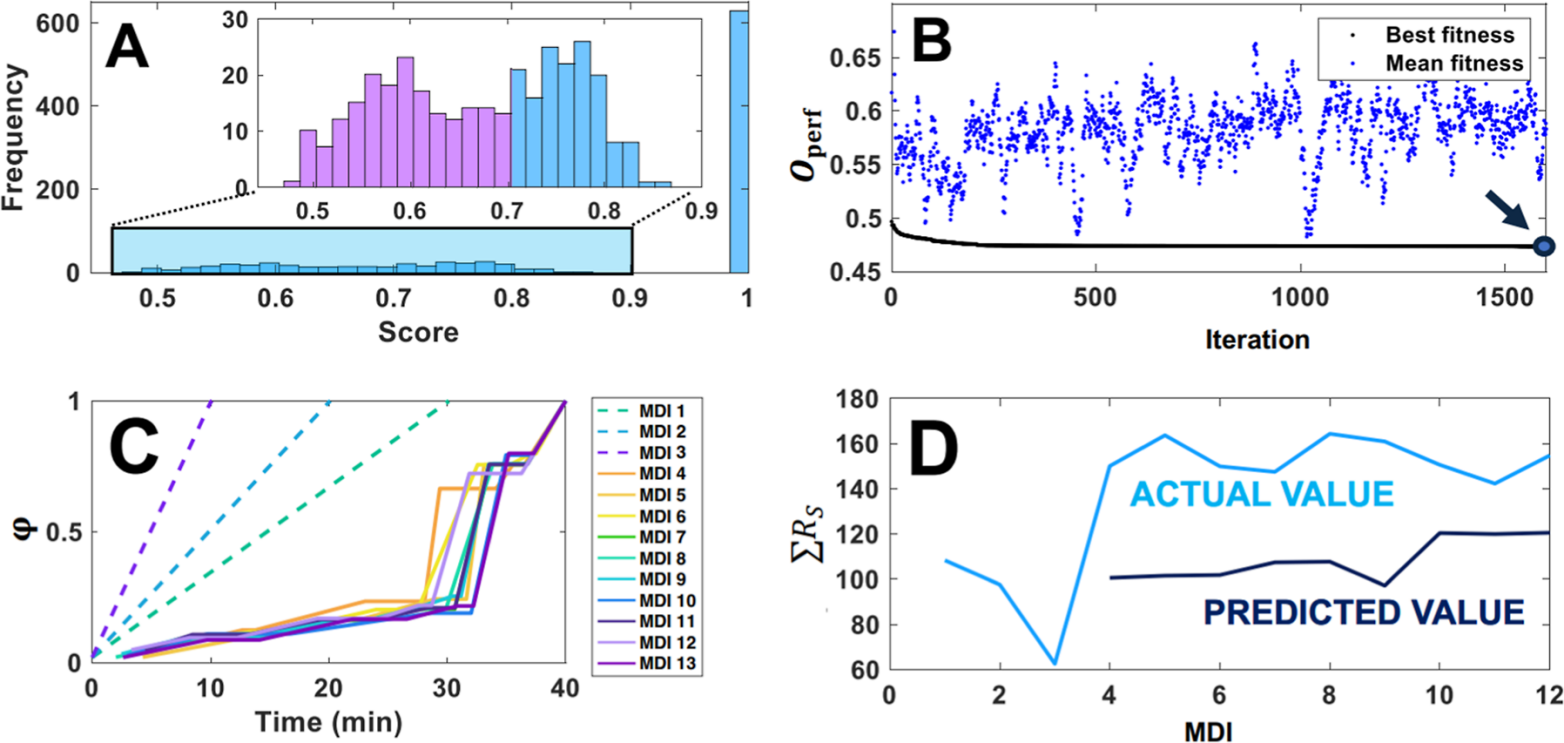
(A) Overview of performance assessment for a representative set of 2000 candidate gradients simulated within one MDI in phase V. Purple bars depict the 200 best (minimum ) candidate gradients, which are further optimized. (B) Mean  for the population of 200 candidate gradients as a function of the genetic algorithm iterations (blue dots) and the best value encountered thus far among all iterations (black line). Arrow depicts the optimal candidate programmed in the next MDI. (C) Optimized gradient used for each experimental MDI (solid lines) and the scouting gradients (dashed lines). (D) ∑*R*_S_ (light blue) and predicted value (dark blue) for each experimental MDI. Data obtained using system A with sample A.

A representative result set for 2000 different optimized gradient programs calculated from a single MDI is shown in Figure 3A. As expected, due to the allowance of a large range of parameter values, most of these candidate gradients did not yield satisfactory scores. This is reflected in the large number (∼600) of nonideal scores (*i.e.*, ) in Figure 3A and highlights the value of exploring a large number of candidate gradient profiles.

In the second stage of this gradient optimization, a genetic algorithm was employed to fine-tune the top 200 candidates from the original pool of 2000 gradients (Figure 3A, purple bars). Longer gradients generally give rise to better separation. However, following optimization, shorter gradients in time result in more efficient methods. To discourage the algorithm from producing very long gradients, a time score, *O*_*t*_, was incorporated in this stage (lower is better) defined by eq 3
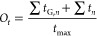
3

A final performance score (*O*_perf_, lower is better) was calculated as a weighted combination of  and *O*_*t*_. Our employed weights were 1.00 for *w*_*R*_s__ and 0.05 for *w*_*t*_, as shown in eq 4. These weights were chosen so that the algorithm would prioritize the resolution before the time score would be significant.
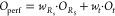
4

Figure 3B displays how the performance score improved as the pool of the 200 top candidates progressed through roughly 1500 iterations of the genetic algorithm. The blue dots depict the mean (*i.e.*, ) score for the 200 candidates in each iteration of the genetic algorithm, and the black solid line reflects the best value obtained for any of the previous iterations. In the terminology of genetic algorithms, the objective criteria (in this case *O*_perf_) is referred to as fitness. From the resulting 200 optimized gradient programs, the best was selected and used for the next MDI (Figure 3B, arrow).

The optimized gradients resulting from the two-stage optimization described in the preceding section, as well as the scouting gradients used to initiate the algorithm, are shown in Figure 3C. Using the retention models, the algorithm tried to employ the first gradient segments to optimize the bulk of the analyte separation observed in the first linear scouting run (Figure 2A, MDI 1) and consistently employed the second last gradient segment to close the gap between analyte distributions. Figure 3D displays the predicted and achieved ∑*R*_S_ of resolution values found for all MDI. MDI 1 through 3 are predetermined scouting gradients, and MDI 4 is the first gradient programmed by the algorithm. The first observation here is that the algorithm immediately proposed a method that appears to achieve an improvement in optimization within the limits of the sampled optimization space. Indeed, further iterations at the best yield limited further improvement. This was expected as the algorithm is designed to leverage the strength of retention modeling, the ability to describe retention as a function of method parameters as opposed to purely exploratory methods such as ML (see below).

The second observation from Figure 3D is that the predicted value is consistently lower than what was achieved. This was found to be largely affected by prediction errors in retention time and peak shape, which significantly improved after the first five MDI (see Supporting Information Section S6). We have implemented a minimum peak width during the prediction of 0.3 min at the base. We did this to ensure a safety margin (*i.e.*, the algorithm would never overestimate the separation). We have provided a table with one example of predicted and experimental widths in Supporting Information Section S7. In addition, there is a possibility that compounds in the sample are not being detected or tracked successfully, leading to an incomplete model. The errors were not found to significantly affect the ability of the algorithm to conduct the optimization. With respect to the number and design of scouting gradients, the optimum number of scouting gradients is likely compound-dependent, and thus by having a standard set of initial gradients that sample the complete φ-range, the workflow remains more general. Our findings here, as well as in Supporting Information Section S8, do not seem to suggest that more than three scouting gradients are necessary.

To further investigate the impact of the availability of data points (*i.e.*, retention times from previous MDI) on the robustness of the retention modeling, we also investigated the optimization of a simple mixture with a unique UV–vis-based peak tracking algorithm.^37^ Here, the quadratic model for the dependence of retention on solvent strength was employed, and we found that for analytes that were difficult to track, up to 10 MDIs were needed to get the retention model to converge. However, this could also be intrinsic to the use of UV–vis data for peak tracking, which was not found to be very robust. Readers interested in further discussion on this aspect of the work are referred to Supporting Information Sections S8 and S9.

### BO of Chromatographic Separations

An alternative to the use of retention models to find candidate gradient elution profiles is to use ML, which is extremely attractive for method development workflows as it does not rely on retention models and thus also does not require peak tracking. Most ML tools require a volume of data that would be considered impractical in order to be functional in the context of chromatographic method development. However, the BO approach generally requires less data to be functional.^38^ We have earlier investigated BO for use in 2D-LC and found it potentially interesting as simulations involving a limited number of iterations were sufficient.^39^ To demonstrate the flexibility of our workflow illustrated in Figure 1, we have also carried out the workflow using BO.

**Figure 4:**
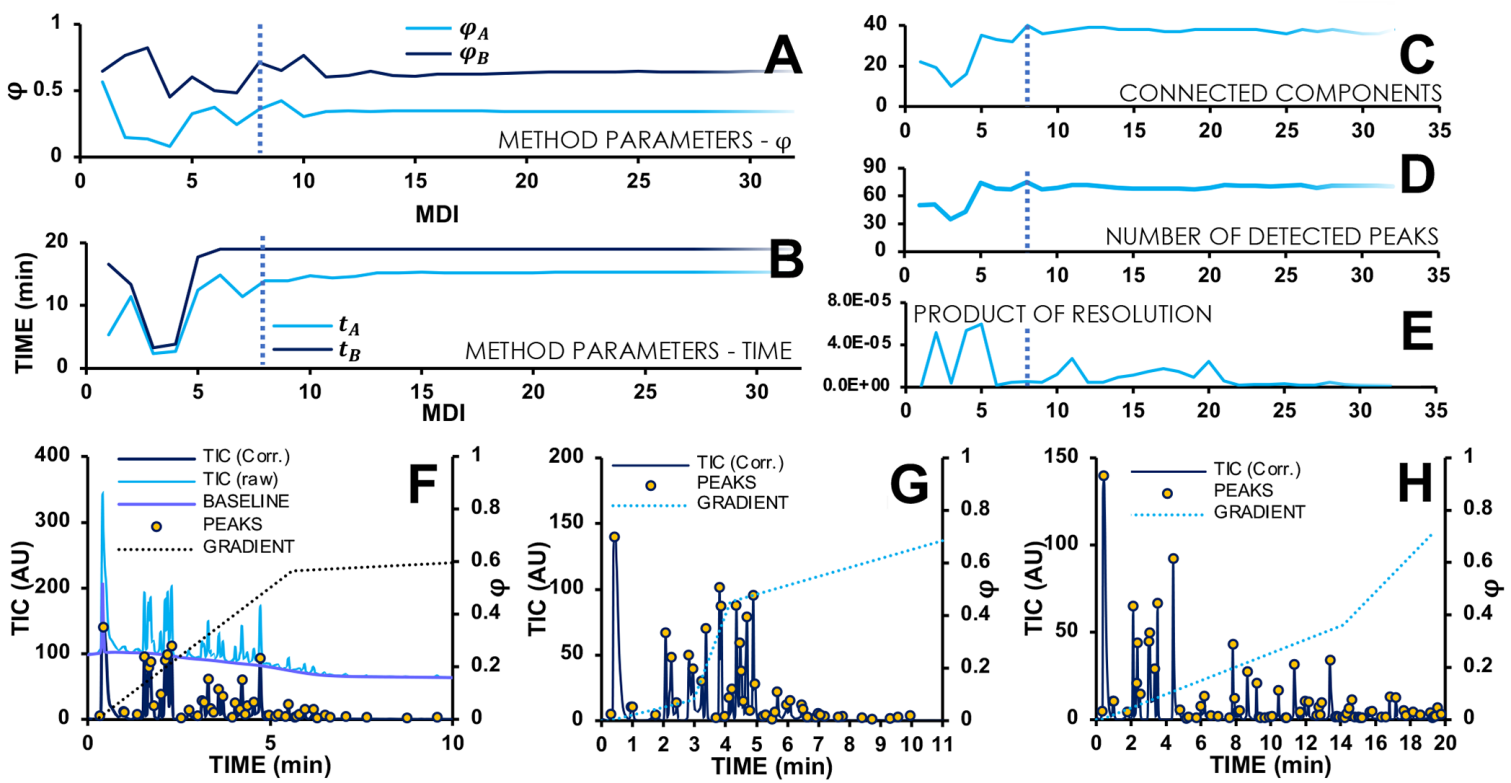
Overview of results using BO. (A,B) Used values for φ_A_ and φ_B_ (A) and *t*_A_ and *t*_B_ (B) as a function of iteration number. (C–E) Observed score according to the number of connected components (C), reweighted resolution (D), and product of resolution (E) as a function of iteration number. In (A–E), the dashed line indicates optimal MDI 8. (F–H) Chromatograms of iterations 1 (F), 4 (G), and 8 (H) in dark blue. Dotted line indicates the gradient program; yellow dots indicate the detected peaks. For panel (F), the raw signal (light blue) and fitted baseline (purple) are also indicated. Data measured on system B with sample B.

In this case, the algorithm was restricted to the development of three-step gradients that always started at a pre-set φ_init_ at *t*_init_ = 0.25 min, and then progressed to φ_A_ at *t*_A_, to φ_B_ at *t*_B_, and ended at a pre-set φ_final_ at *t*_final_ (see Supporting Information Section S10 for a graphical depiction of this gradient). This means that the algorithm was, in fact, optimizing φ_A_, φ_B_, *t*_A_, and *t*_B_. All operations of this algorithm were run in a Python programming environment. The results are shown in Figure 4.

The impressive efficiency of BO becomes immediately apparent in Figure 4. Panels A and B show the ranges of φ_A_ and φ_B_ (Figure 4A), and *t*_A_ and *t*_B_ (Figure 4B) explored by the algorithm in each MDI. After about 8 MDIs, the values stabilize, suggesting that the algorithm converges to an optimum. This is supported by the score plot in Figure 4C, where the algorithm assesses the number of connected components^39^ (Figure 4C). It can be seen that this number approximates the actual number of detected peaks (Figure 4D). In 1D-LC, the number of connected components amounts to the sum of the number of (unseparated) peak clusters (*R*_s_ < 1.5) and the number of separated peaks. The importance of the features of the objective function becomes apparent in Figure 4E, where the product of resolution (all obtained resolution values between all adjacent peaks multiplied with each other) is used similar to our earlier 2D optimization work.^15^ Relative to the first experiment (Figure 4F, MDI 1, 22 connected components, 50 detected peaks), the optimum according to the product of resolution function (Figure 4G, MDI 5, 34 connected components, 73 detected peaks) does not yield the same number of separated species as the optimum according to the score functions of connected components and detected peaks (Figure 4H, MDI 8, 40 connected components, 75 detected peaks).

It is important to note that, different from the retention modeling strategy, there were just four parameters optimized in this BO study. It is expected that increasing the dimensionality of this gradient to reach the complexity of the sophisticated gradient used in combination with the retention modeling approach would increase the number of MDIs needed to converge to an optimum. Nevertheless, the present example demonstrates the potential of BO for method optimization and we will investigate this further in the future.

## Roadmap for Future Development

The algorithm and its implementations described here by no means represent a final solution; there is plenty of room for future improvement. Arguably, both implementations (retention modeling, BO) exhibit attractive characteristics for use in unsupervised, automated method development. While the complexities of the samples used for these two cases were different, the strengths and weaknesses of several operations for each implementation became apparent. Based on these observations, we identified key areas that future research should focus on.

In any method development process, the decision to continue requires a careful cost-benefit balance. This is also true for our workflow, where there is an experimental and computational cost associated with continuing with each strategy that is mainly expressed through the number of required iterations to reach an optimum and the length of each iteration. In the present study, the ML strategy required approximately 10 MDIs to optimize four parameters, relative to the 4–5 MDIs required to optimize 16 parameters when using the retention modeling strategy. However, the performance of retention modeling strongly depends on the success of peak tracking, prediction of separations, and fitting an accurate retention model. This is very different from BO, where there is no such dependence, yet more MDI are required to map the relation between chromatographic parameters and the objective function score. In addition, the quality and robustness of the objective function are crucial to the effectiveness of BO. This will be of significant relevance for the application of this workflow for 2D-LC, where the number of parameters is doubled, and thus, the search space grows exponentially due to the interdependence of the first- and second-dimension parameters.

Finally, the objective function quantifies the goal of method development and drives the optimization process. Traditional objective functions quantify the performance of the separation method using quality descriptors such as peak capacity, or orthogonality in 2D separations. However, as can be seen in Figure 4C–E, maximization of such descriptors does not necessarily lead to a better separation. There is a need for chromatographic response functions that comprehensively summarize quality descriptors such as resolution and peak capacity and also quantify the practical component of an analytical question.

### Signal Processing: Background Correction, Peak Detection, and Peak Tracking

The performance of an algorithm is likely to improve when provided with better input data. For either strategy, this is certainly true; one mistake can result in a cascade reaction (*i.e.*, background correction anomalies affecting retention modeling or the optimization process later on). This already starts with background correction and peak detection, which is critical for all optimization strategies. The use of MS simplifies this problem for 1D separations. Nevertheless, quantitative performance comparisons of data processing algorithms barely exist, in particular not for multivariate data,^18^ and we found that it is difficult to rely on a single algorithm since these often depend on signal characteristics. One solution may be the Autoencoder which was recently developed^40^ and shown to be rather robust.^19^

Peak detection is another focus point, which is important in phase II of any implementation of the algorithm because it supplies the number of analyte peaks to separate and also drives the peak tracking process. The latter is particularly crucial when using retention modeling in phase IV. Unsurprisingly, we find that the use of a mass spectrometer almost appears mandatory, with the UV–vis peak tracking exclusively useful for mixtures of compounds with distinct spectra such as the dye mixture used in this work.

### Retention Modeling and Gradient Deformation

The use of retention data from gradient elution experiments rather than isocratic measurements to construct retention models has been a point of concern.^41^ For automated method development, we find our results encouraging. Looking forward to method development for applications that utilize fast separations (*e.g.*, 2D-LC), we are concerned about gradient deformation when steep gradients are utilized.^35^ Nevertheless, our current data suggests that the deformation of the used gradients was minimal (see Supporting Information Section S11). However, when a less advanced LC pump is used in combination with steep gradients or low flow rates, this deformation may have an influence on the retention model and prediction of retention times.

## Conclusions and Outlook

We have developed and demonstrated an interpretive algorithm that is capable of unsupervised, automated method development for LC separations. Based on our findings, we draw the following conclusions:

- Our workflow allows unsupervised method development and facilitates complete automation from executing the scouting gradients all the way through obtaining fine-tuned methods.
- The use of retention modeling appears to quickly (<5 MDIs) yield useful improvements over the initial scouting gradients when optimizing a sophisticated gradient program (16 correlated parameters). This approach, however, heavily relies on peak detection and tracking.
- We find BO promising for the optimization of chromatographic methods. While our assessment in this study only tasked BO with the optimization of four parameters, we found that BO indeed offers rapid improvements (8–10 MDIs) without relying on knowledge from prior experiments (*e.g.*, peak tracking). We also see the potential for BO in other analytical optimization tasks as long as an accurate objective function can be defined.
- We envisage further opportunities for extending automation to include selectivity screening, 2D-LC, and kinetic optimization.

We do not feel that the algorithm discussed here represents a finished product, and we thus have proposed areas to focus on in subsequent work. While the algorithm technically does not require information about the sample, the user still must decide on the stationary phase selectivity and kinetic parameters (*e.g.*, flow rate). We believe that this is fair because if one knows the sample type (*e.g.*, peptides), this dictates column chemistry. Column dimensions and flows are determined by analysis time and can be estimated using determined using tools such as kinetic plots.^6^ To allow the community to benefit and improve this work, a prototype version of the algorithm is shared in the Supporting Information in Section S12.

## Supporting Information Available

Additional data supporting the samples, LC–MS peak tracking, retention modeling constraints, employed gradient programs, score function visualizations, UV–vis peak tracking, gradient deformation, predicted chromatograms, and the algorithm as well as further design considerations (PDF)
Algorithm code used in this work (ZIP)
