# Supplementary material for: Chemometric Strategies for Fully Automated Interpretive Method Development in Liquid Chromatography
Source: Anal Chem. 2022 Nov 1;94(46):16060–8. doi: 10.1021/acs.analchem.2c03160 (PMC9685588; doi:10.1021/acs.analchem.2c03160)
Supplement: Supplementary file 1 — ac2c03160_si_001.pdf [file ac2c03160_si_001.pdf]

SUPPORTING INFORMATION

Chemometric Strategies for Fully Automated Interpretive Method Development in Liquid Chromatography

Tijmen S. Bos[a,d,1], Jim Boelrijk[b,d,e,1], Stef R.A. Molenaar[c,d,1], Brian van ‘t Veer[c,d], Leon E. Niezen[c,d], Denice van Herwerden[c,d], Saer Samanipour[c,d], Dwight R. Stoll[g], Patrick Forré[b,e], Bernd Ensing[e,f], Govert W. Somsen[a,d], Bob W.J. Pirok[c,d,e,g,*]

[a] Division of Bioanalytical Chemistry, Amsterdam Institute of Molecular and Life Sciences, Vrije Universiteit Amsterdam, De Boelelaan 1085, 1081HV Amsterdam, The Netherlands

[b] AMLab, Informatics Institute, University of Amsterdam, Science Park 904, 1098 XH Amsterdam, The Netherlands

[c] Analytical Chemistry Group, Van ’t Hoff Institute for Molecular Sciences, University of Amsterdam, Science Park 904, 1098XH Amsterdam, The Netherlands

[d] Centre for Analytical Sciences Amsterdam (CASA), Science Park 904, 1098XH Amsterdam, The Netherlands

[e] AI4Science Lab, University of Amsterdam, Science Park 904, 1098XH Amsterdam, The Netherlands

[f] Computational Chemistry Group, Van ‘t Hoff Institute for Molecular Sciences, University of Amsterdam, Science Park 904, 1098XH Amsterdam, The Netherlands

[g] Department of Chemistry, Gustavus Adolphus College, Saint Peter, MN 56082, United States

[1] Equal contributions

(*) Corresponding author

E-mail: B.W.J.Pirok@uva.nl

Contents

S-1 Samples.. S-3
S-2 Peak tracking results for LC-MS measurements of antibody digest sample S-4
S-3 Retention modeling constraints S-15
S-4 Overview of gradient program...S-15
S-5 Predicted score function S-16
S-6 Prediction errors in retention time and peak width S-16
S-7 Minimum peak width S-18
S-8 In-depth study of retention modeling using UV-vis data and the quadratic model S-21
S-9 UV-Vis peak tracking data S-23
S-10 Gradient design of Bayesian Optimization S-25
S-11 Gradient deformation S-25
S-12 Algorithm S-26
References...S-26

S-1 Samples

Sample A was an antibody digest dissolved in buffer with an earlier reported minimum number of 189 compounds[1].

For Bayesian optimization, Sample B was a mixture of 80 dye reference compounds was obtained from the Dutch Cultural Heritage Agency, and used and prepared as reported earlier[2]. Sample B contained at least 158 components. To prepare the sample mixture for retention modelling, all dyes for Sample B were dissolved at a concentration of 50 ppm in eluent B.

S-2 Peak tracking results for LC-MS measurements of antibody digest sample

These are the peak tracking results for the optimization of the LC-MS of Sample A (antibody digest) on System B. The first item is the peak tracking table.

Table S-1. Peak tracking results for optimization of LC-MS separation of antibody digest sample (Sample A) on System B.

Peak #	t_R (MDI 1)	tt_R (MDI 2)	t_R (MDI 3)	t_R (MDI 4)	t_R (MDI 5)	t_R (MDI 6)	t_R (MDI 7)	t_R (MDI 8)	t_R (MDI 9)	t_R (MDI 10)	t_R (MDI 11)	t_R (MDI 12)
**1**	0.686	0.689	0.686	0.691	0.688	0.695	0.690	0.684	0.691	0.690	0.688	0.689
**2**	0.797	0.783	0.791	0.724	0.777	0.684	0.701	0.778	0.729	0.745	0.699	0.700
**3**	1.007	1.121	1.134	1.034	1.086	0.772	0.817	1.076	1.056	1.077	0.826	0.816
**4**	1.018	1.441	1.466	14.371	1.368	1.032	1.044	1.336	1.177	1.253	1.041	0.927
**5**	1.023	1.734	1.598	1.305	6.398	1.038	1.044	2.143	1.503	4.183	1.064	1.043
**6**	1.029	1.977	1.715	2.090	5.049	1.176	1.342	4.304	2.305	3.017	1.412	1.291
**7**	1.023	2.104	1.775	2.758	2.203	1.309	1.049	4.890	3.173	1.723	1.705	1.048
**8**	1.029	2.298	1.902	3.521	6.823	1.778	2.216	5.277	3.946	4.901	2.208	2.120
**9**	1.029	2.298	1.902	7.164	9.570	1.778	4.592	7.184	6.113	7.029	2.208	2.120
**10**	2.582	2.701	2.090	7.473	9.830	3.298	4.813	7.322	6.235	7.173	4.667	4.403
**11**	2.704	2.889	2.124	8.374	10.648	4.155	6.361	7.941	6.815	7.742	6.215	5.597
**12**	2.527	2.618	2.007	8.883	11.096	4.973	7.400	8.190	7.047	7.947	7.149	6.382
**13**	2.797	2.790	2.107	8.883	11.124	5.028	7.212	8.295	7.152	8.057	7.011	6.288
**14**	3.035	2.889	2.444	8.816	11.096	5.272	7.091	8.377	7.252	8.146	6.922	6.288
**15**	2.753	2.679	2.019	9.612	11.969	7.809	8.484	8.897	7.655	8.505	7.873	7.482
**16**	1.023	2.790	2.074	9.684	12.030	7.576	8.484	8.980	7.749	8.610	7.939	7.482
**17**	3.317	3.144	2.345	9.684	12.030	6.963	8.218	9.157	7.954	8.831	7.873	7.338
**18**	3.079	2.889	2.124	10.336	12.832	24.639	9.263	27.176	8.318	9.141	8.465	8.294
**19**	4.876	NaN	NaN	NaN	NaN	NaN	NaN	NaN	8.318	NaN	NaN	NaN
**20**	3.925	3.530	2.571	11.055	13.578	9.096	9.827	10.417	9.109	9.942	9.034	8.836
**21**	3.494	3.127	2.251	11.397	14.130	10.755	10.413	10.760	9.280	10.069	9.260	9.494

**22**	3.715	3.293	2.367	11.464	14.130	10.506	10.413	10.832	9.380	10.185	9.360	9.494
**23**	3.853	3.398	2.439	11.580	14.235	10.506	10.506	10.953	9.512	10.324	9.470	9.588
**24**	3.853	NaN	NaN	NaN	NaN	NaN	NaN	NaN	9.512	NaN	NaN	NaN
**25**	3.737	3.238	2.300	11.983	14.877	11.821	11.186	11.395	9.833	10.578	9.675	10.522
**26**	4.152	3.542	2.510	12.337	15.170	11.821	11.557	11.766	10.336	10.976	10.095	10.820
**27**	4.030	3.464	NaN	12.409	15.391	12.225	NaN	11.887	10.441	NaN	NaN	NaN
**28**	4.224	3.613	2.549	12.520	15.391	12.037	11.850	11.987	10.585	11.142	10.294	11.135
**29**	4.423	3.724	2.599	12.890	15.971	12.595	12.607	12.562	11.220	11.545	10.758	11.998
**30**	4.196	3.464	2.405	13.094	16.883	13.585	15.453	13.662	12.718	11.827	11.223	14.761
**31**	4.804	3.951	2.732	13.713	17.231	13.822	14.779	14.170	13.033	12.756	12.135	14.286
**32**	4.318	3.530	2.433	13.332	17.209	13.944	16.150	14.601	13.884	12.341	11.869	15.176
**33**	4.550	3.558	2.510	13.708	17.767	14.568	16.841	16.254	16.001	13.513	13.351	15.729
**34**	4.887	3.917	2.671	14.355	18.265	15.171	17.194	16.790	16.543	14.674	14.528	16.099
**35**	4.876	NaN	NaN	NaN	NaN	NaN	NaN	NaN	16.543	NaN	NaN	NaN
**36**	4.710	3.785	2.571	14.205	20.741	15.215	17.366	17.083	17.096	14.967	14.970	16.204
**37**	5.042	3.846	2.709	15.057	18.834	8.969	9.263	9.638	17.825	16.884	16.031	8.294
**38**	4.865	3.846	2.599	14.786	18.696	16.343	17.891	17.835	17.958	17.470	16.170	16.696
**39**	5.235	4.056	2.709	15.311	18.994	17.282	18.206	18.177	18.350	21.058	16.667	16.989
**40**	2.864	2.790	2.074	16.190	12.030	7.576	8.484	18.841	19.008	8.610	17.374	7.482
**41**	5.489	3.912	2.848	16.339	19.840	18.487	18.958	18.951	19.102	20.311	17.496	17.751
**42**	5.235	4.056	2.632	16.190	19.680	18.686	18.886	18.951	19.191	21.058	17.607	17.641
**43**	5.578	4.315	2.875	16.593	20.067	18.952	19.245	19.216	19.406	20.925	17.817	18.011
**44**	5.417	4.183	2.825	16.599	20.067	19.128	19.273	19.332	19.583	21.671	18.032	18.011
**45**	5.495	4.260	2.776	16.654	20.072	19.090	19.295	19.355	19.605	21.455	18.005	18.011
**46**	1.007	4.188	2.770	7.363	9.476	1.662	3.039	6.736	19.616	6.654	2.921	2.612
**47**	5.528	4.227	3.439	16.936	20.393	19.565	19.544	19.697	19.964	22.412	18.430	18.299
**48**	5.583	4.564	2.798	17.428	20.923	20.051	19.997	20.084	20.384	23.186	18.894	18.785
**49**	5.931	NaN	NaN	NaN	NaN	NaN	NaN	NaN	20.384	NaN	NaN	NaN
**50**	5.755	4.371	NaN	17.776	21.283	20.637	20.395	20.620	20.965	24.053	19.497	19.128

**51**	5.755	4.315	NaN	18.168	21.747	21.157	20.815	21.107	21.512	25.137	20.094	19.537
**52**	6.241	4.675	3.019	19.014	22.935	22.058	21.976	22.212	22.446	26.170	21.116	20.438
**53**	6.562	4.907	3.157	19.473	23.786	22.522	22.838	22.969	23.054	26.668	21.807	21.090
**54**	6.562	4.907	3.229	19.694	24.378	22.810	23.661	23.732	23.596	27.204	22.537	21.549
**55**	6.783	5.078	3.257	19.998	24.715	23.114	24.220	24.268	24.104	27.502	23.056	22.079
**56**	6.562	5.078	3.229	20.363	25.367	23.556	25.977	25.694	25.508	28.293	24.858	23.329
**57**	6.904	5.144	3.284	20.462	25.461	23.672	26.126	25.838	25.779	28.420	25.151	23.572
**58**	6.982	5.277	3.257	20.727	25.721	23.948	26.408	26.103	26.254	28.713	25.627	24.135
**59**	7.081	NaN	NaN	NaN	NaN	NaN	NaN	NaN	26.254	NaN	NaN	NaN
**60**	7.147	5.343	NaN	20.932	25.970	24.180	26.801	26.463	26.724	29.056	26.268	24.904
**61**	7.241	5.410	3.417	21.253	26.373	24.562	27.392	27.004	27.349	29.652	27.152	26.269
**62**	7.241	5.377	3.389	21.938	27.153	24.639	27.580	27.170	27.537	29.840	27.467	26.617
**63**	7.396	5.531	3.478	21.722	26.893	25.092	28.044	27.651	28.012	30.542	28.097	27.242
**64**	7.490	5.377	3.389	21.308	26.478	25.363	28.376	27.972	28.316	31.344	28.639	27.701
**65**	7.490	5.780	3.389	22.032	26.478	25.490	28.503	28.093	28.427	31.510	28.733	27.772
**66**	7.628	5.343	3.389	22.120	27.423	25.866	28.835	28.408	28.681	31.504	29.109	28.209
**67**	7.811	6.051	3.599	22.690	27.915	26.529	29.404	28.922	29.057	33.383	29.584	28.474
**68**	7.501	NaN	NaN	NaN	NaN	NaN	NaN	28.834	29.062	NaN	NaN	NaN
**69**	7.490	5.437	3.334	23.093	28.341	28.934	32.024	31.327	29.460	33.394	32.005	29.077
**70**	7.966	5.868	3.671	23.215	28.457	27.856	30.642	30.000	29.582	33.560	30.529	29.077
**71**	7.628	5.531	3.367	23.486	28.695	29.006	32.024	31.327	29.764	33.394	32.005	29.994
**72**	8.209	5.587	3.417	23.751	29.004	29.006	32.024	31.327	29.991	33.472	32.005	29.994
**73**	7.750	NaN	NaN	NaN	NaN	NaN	NaN	NaN	29.991	NaN	NaN	NaN
**74**	7.883	5.669	3.439	24.116	29.314	29.044	32.024	31.327	30.278	33.472	32.005	29.994
**75**	8.562	6.189	3.754	26.581	31.005	29.315	32.195	31.537	31.411	33.621	32.171	30.204
**76**	8.772	6.394	3.793	29.416	32.917	29.315	32.195	31.537	32.235	33.621	32.171	30.204
**77**	8.861	NaN	NaN	NaN	NaN	NaN	NaN	NaN	32.235	NaN	NaN	NaN
**78**	8.772	6.300	3.793	29.339	32.829	29.315	32.195	31.537	32.323	33.621	32.171	30.276
**79**	9.021	6.598	3.876	29.416	32.917	29.426	32.251	31.620	32.467	33.671	32.226	30.276

**80**	9.209	NaN	NaN	NaN	NaN	NaN	NaN	NaN	32.467	NaN	NaN	NaN
**81**	9.364	7.024	4.102	29.416	32.917	29.625	32.400	31.802	32.561	33.803	32.370	30.431
**82**	9.679	NaN	NaN	NaN	NaN	NaN	NaN	NaN	32.561	NaN	NaN	NaN
**83**	13.189	9.693	5.910	30.201	33.702	31.261	33.533	33.178	33.595	34.848	33.481	31.697
**84**	19.943	12.965	7.844	31.467	34.598	33.063	34.760	34.676	34.711	35.959	34.686	33.079
**85**	17.428	NaN	NaN	NaN	NaN	NaN	NaN	NaN	34.711	NaN	NaN	NaN
**86**	21.402	15.497	9.104	33.716	35.637	34.350	35.843	35.815	35.789	36.799	35.769	34.510
**87**	21.220	16.133	9.447	34.844	36.184	34.898	36.385	36.351	36.331	37.219	36.311	35.212
**88**	22.226	16.586	9.663	35.778	36.588	35.329	36.789	36.754	36.740	37.512	36.720	35.798
**89**	23.000	NaN	NaN	NaN	NaN	NaN	NaN	NaN	36.740	NaN	NaN	NaN
**90**	23.000	NaN	NaN	NaN	NaN	NaN	NaN	NaN	36.740	NaN	NaN	NaN
**91**	22.182	NaN	NaN	NaN	NaN	NaN	NaN	NaN	36.740	NaN	NaN	NaN
**92**	25.012	17.393	9.519	38.221	37.350	36.445	37.618	37.561	37.486	37.219	37.510	38.202
**93**	24.354	17.747	10.232	37.746	38.174	36.976	38.325	38.296	38.293	38.645	38.284	37.937
**94**	24.735	NaN	NaN	NaN	NaN	NaN	NaN	NaN	38.293	NaN	NaN	NaN
**95**	25.940	17.984	9.801	38.868	38.732	38.175	38.800	38.794	38.790	38.435	38.804	38.949
**96**	27.140	19.289	NaN	39.807	39.921	39.883	39.983	39.977	39.990	39.966	39.992	40.015
**97**	27.444	NaN	NaN	NaN	NaN	NaN	NaN	NaN	39.990	NaN	NaN	NaN
**98**	NaN	1.192	1.212	0.995	1.086	0.645	NaN	1.082	1.045	1.071	NaN	NaN
**99**	NaN	1.281	1.294	1.061	1.246	0.772	0.817	1.214	1.072	1.071	1.041	1.020
**100**	NaN	2.442	1.952	6.136	8.957	2.243	1.281	6.537	5.439	6.421	1.323	1.253
**101**	NaN	2.187	1.764	7.363	9.476	19.084	3.039	6.736	5.726	6.654	2.921	18.067
**102**	NaN	3.039	2.212	10.673	13.191	9.306	9.572	9.991	8.617	9.450	8.730	8.632
**103**	NaN	3.962	NaN	NaN	NaN	NaN	NaN	NaN	17.963	NaN	NaN	NaN
**104**	NaN	5.587	3.439	15.775	19.558	18.487	18.670	18.619	18.859	NaN	17.374	17.475
**105**	NaN	4.183	2.737	NaN	NaN	NaN	NaN	NaN	19.594	NaN	NaN	18.011
**106**	NaN	NaN	2.776	NaN	13.445	NaN	NaN	NaN	9.098	NaN	NaN	NaN
**107**	NaN	NaN	2.444	NaN	NaN	NaN	NaN	NaN	13.044	NaN	NaN	NaN
**108**	NaN	NaN	3.141	19.909	24.715	23.114	24.667	24.887	24.237	27.646	23.454	22.251

**109**	NaN	NaN	NaN	1.570	1.644	1.508	1.525	1.645	1.603	1.618	1.544	1.529
**110**	NaN	NaN	NaN	9.059	11.339	5.487	7.395	8.499	7.346	8.240	7.149	6.498
**111**	NaN	NaN	NaN	14.089	18.132	14.889	17.211	8.897	17.007	8.505	16.269	15.977
**112**	NaN	NaN	NaN	20.153	24.942	23.279	24.667	24.699	24.464	27.646	23.454	22.411
**113**	NaN	NaN	NaN	21.308	26.478	NaN	NaN	NaN	28.703	NaN	NaN	27.634
**114**	NaN	NaN	NaN	30.560	34.034	32.073	34.047	33.836	34.064	35.312	33.984	32.283
**115**	NaN	NaN	NaN	NaN	2.916	1.458	1.635	2.867	2.189	2.431	1.649	1.601
**116**	NaN	NaN	NaN	NaN	17.999	14.889	17.211	8.897	16.731	8.505	16.269	15.977
**117**	NaN	NaN	NaN	NaN	NaN	0.656	0.718	0.778	0.757	0.745	0.732	0.728
**118**	NaN	NaN	NaN	NaN	NaN	NaN	28.840	NaN	28.697	NaN	NaN	NaN
**119**	NaN	NaN	NaN	NaN	NaN	NaN	NaN	NaN	0.884	0.960	0.826	0.810
**120**	NaN	NaN	NaN	NaN	NaN	NaN	NaN	NaN	34.274	35.528	34.205	32.520
**121**	NaN	NaN	NaN	NaN	NaN	NaN	NaN	NaN	7.594	NaN	7.553	6.929
**122**	NaN	NaN	NaN	NaN	NaN	NaN	NaN	NaN	2.222	NaN	NaN	2.933
**123**	NaN	NaN	NaN	NaN	NaN	NaN	NaN	NaN	7.290	NaN	NaN	6.968

The next items are the resulting chromatograms for each iteration with peak tracking results. Note that due to the retrack occurring during iterations 4 and 9, chromatograms of the three groups of iteration 1-3, 4-8 and 9-12 each feature different number annotations relative to the other groups.

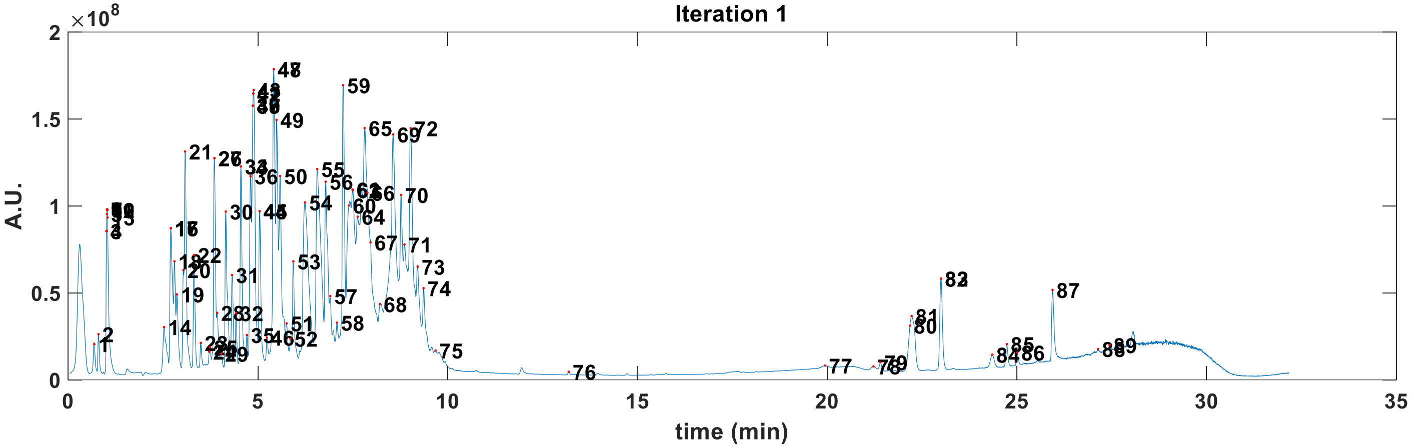

Figure S-1. LC-MS TIC chromatogram of iteration 1.

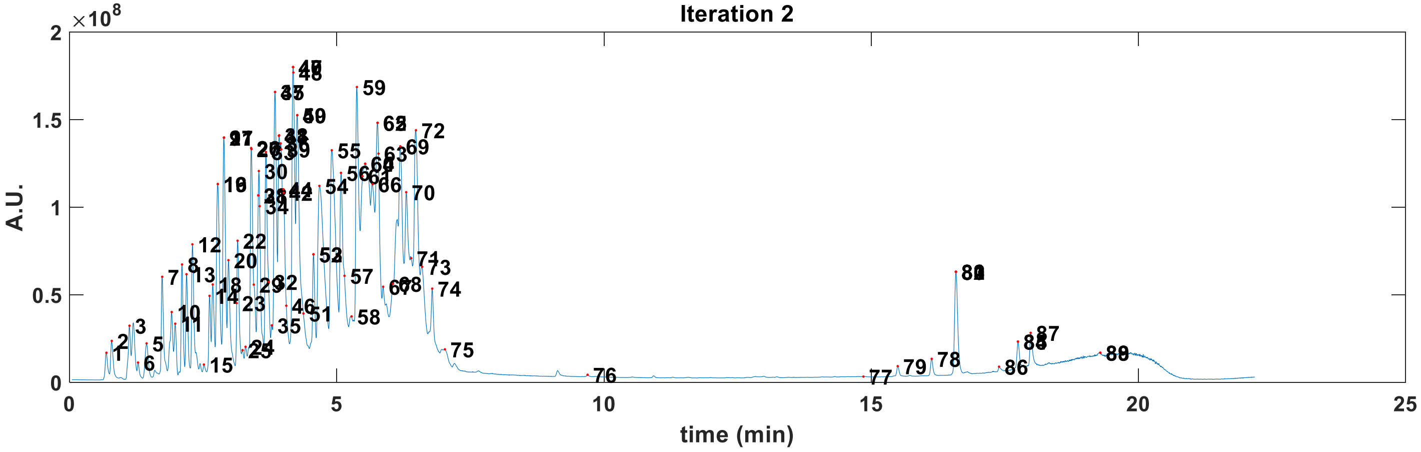

Figure S-2. LC-MS TIC chromatogram of iteration 2.

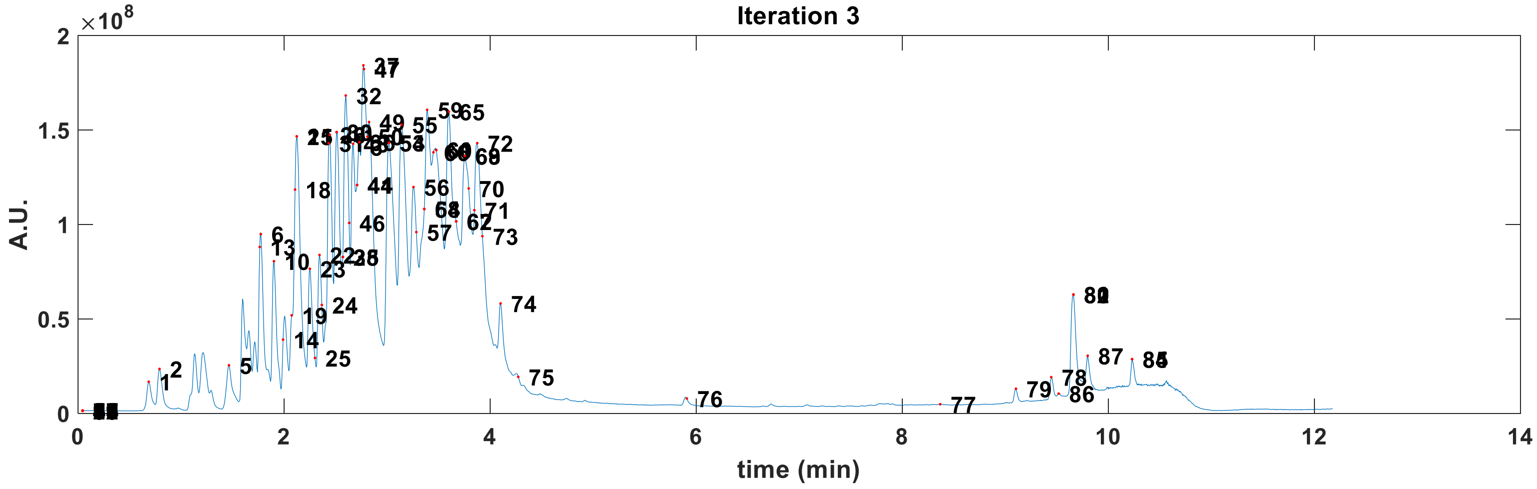

Figure S-3. LC-MS TIC chromatogram of iteration 3.

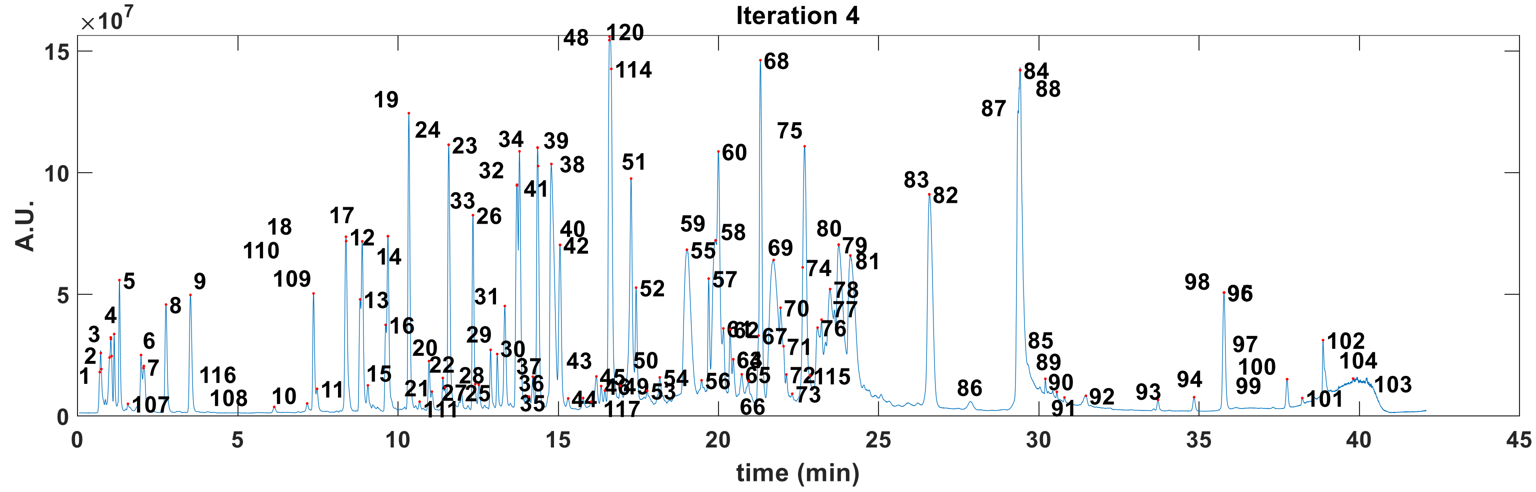

Figure S-4. LC-MS TIC chromatogram of iteration 4.

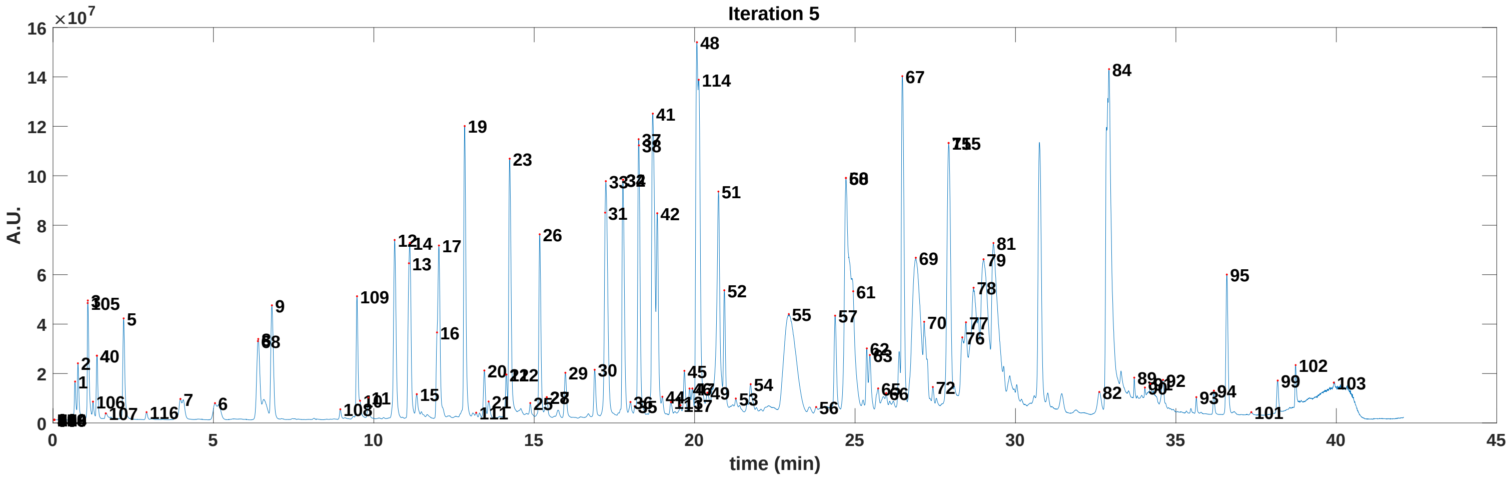

Figure S-5. LC-MS TIC chromatogram of iteration 5.

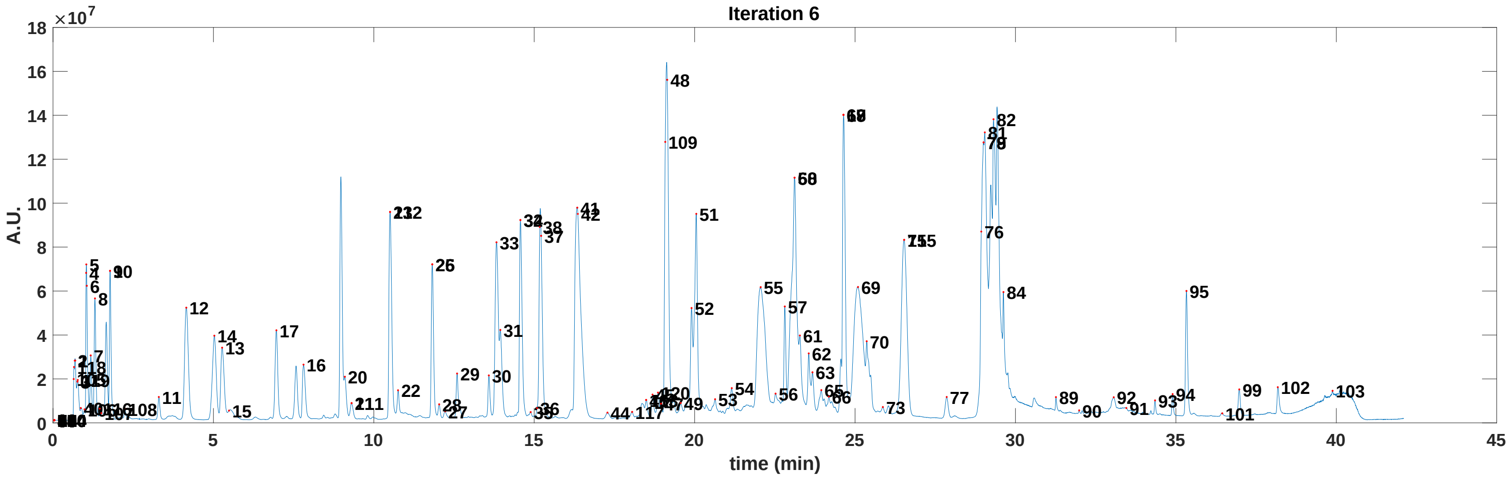

Figure S-6. LC-MS TIC chromatogram of iteration 5.

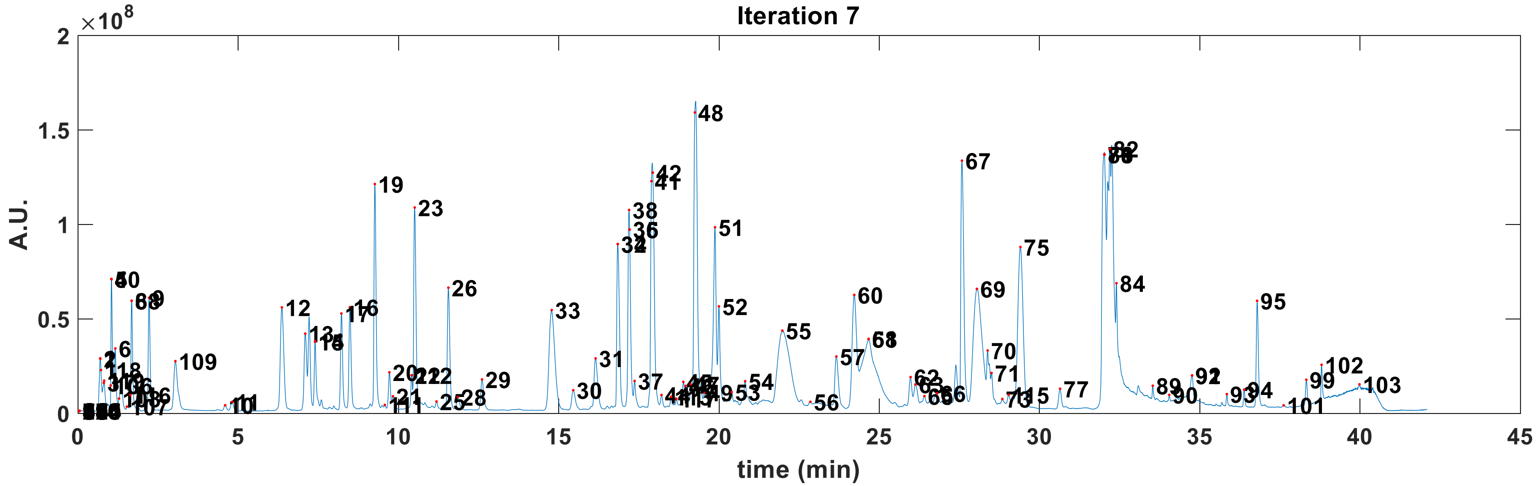

Figure S-7. LC-MS TIC chromatogram of iteration 7.

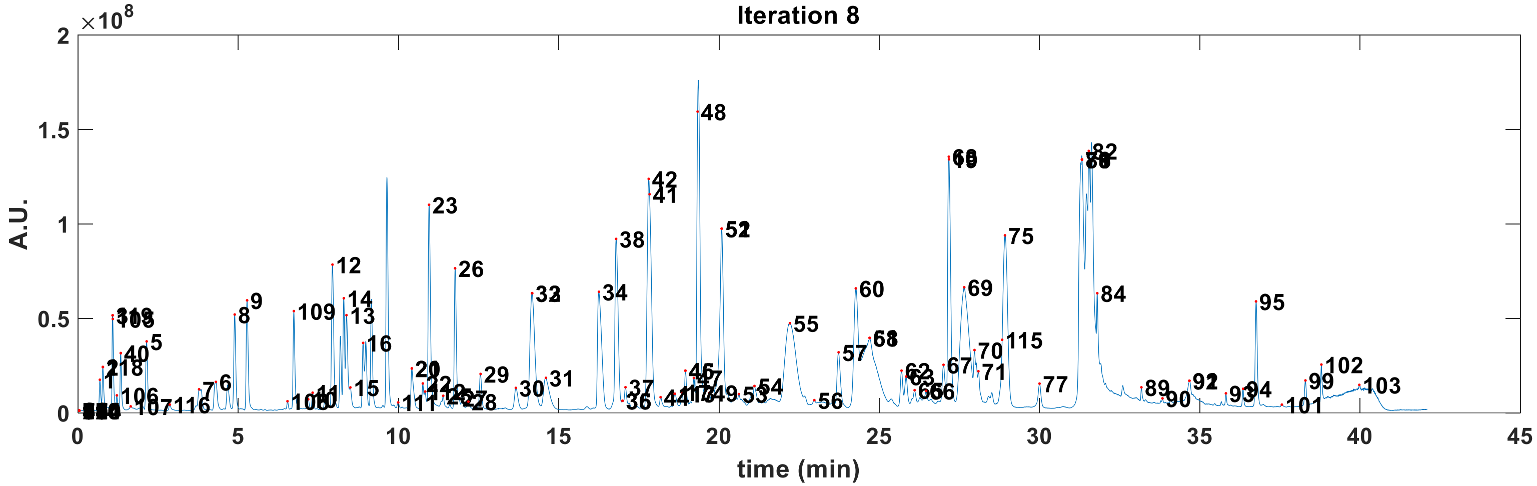

Figure S-8. LC-MS TIC chromatogram of iteration 8.

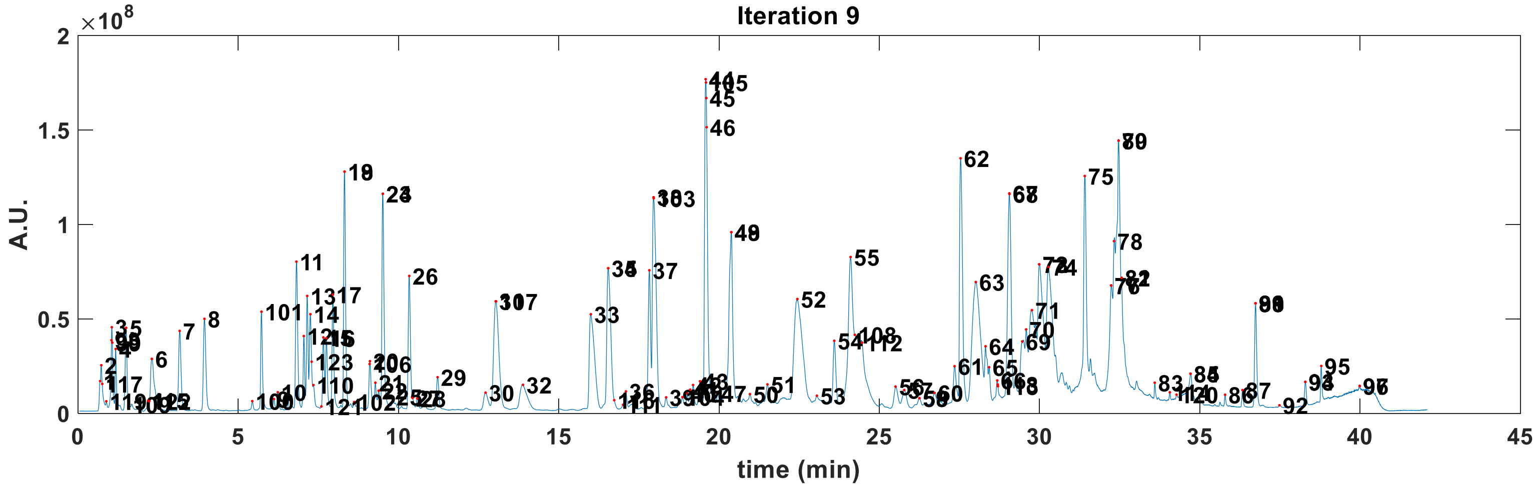

Figure S-9. LC-MS TIC chromatogram of iteration 7.

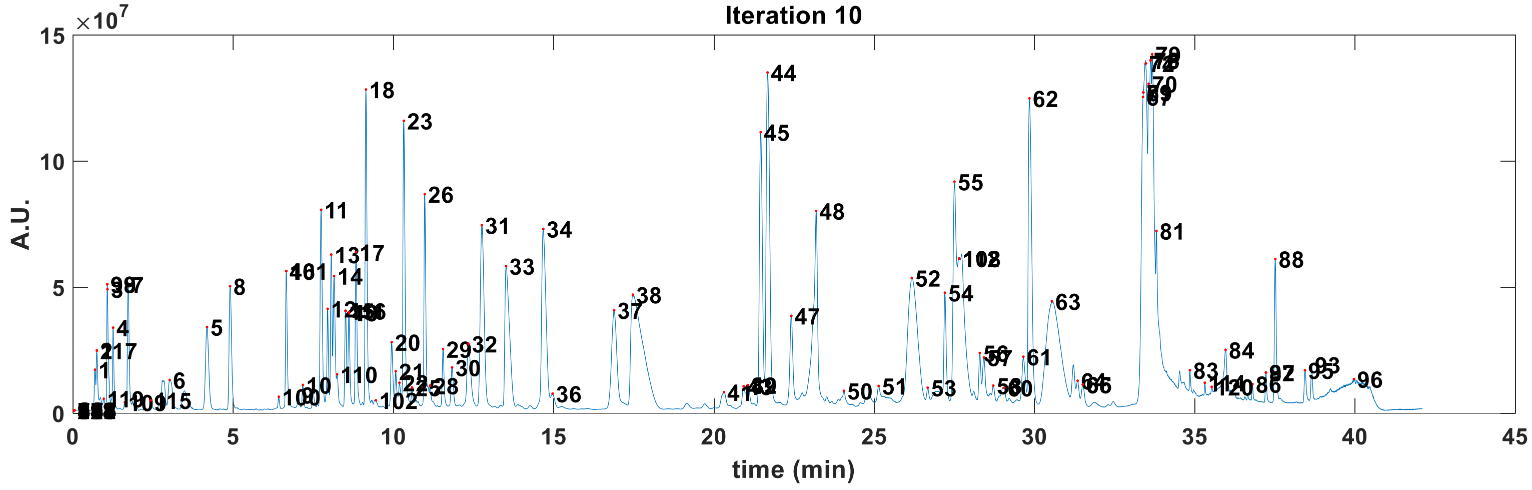

Figure S-10. LC-MS TIC chromatogram of iteration 10.

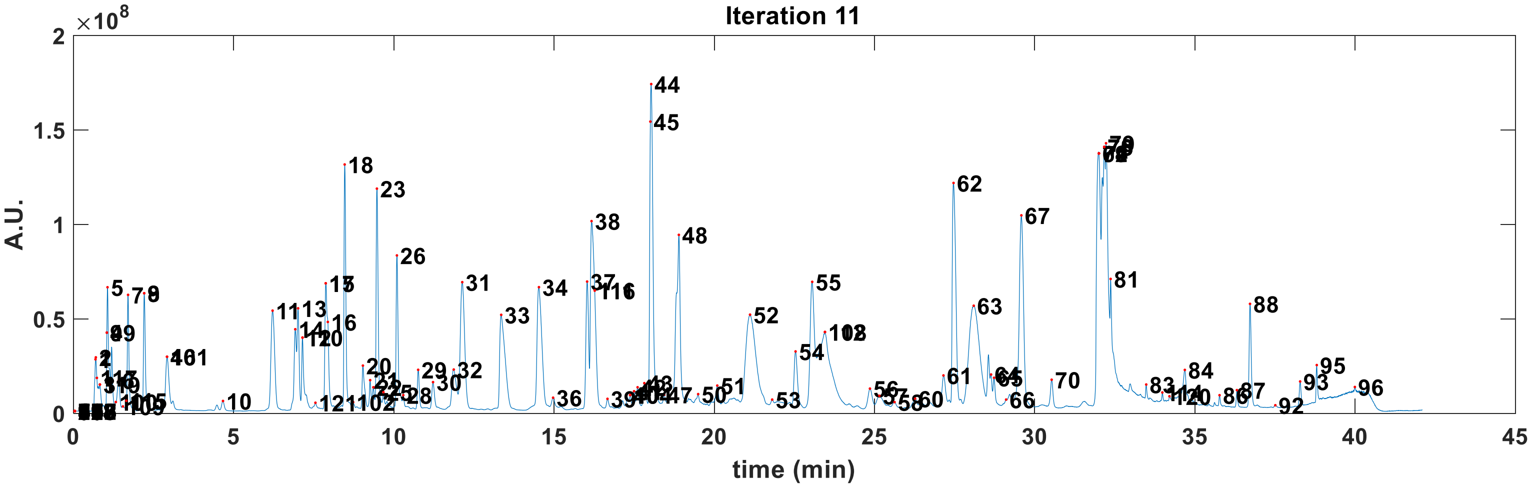

Figure S-11. LC-MS TIC chromatogram of iteration 11.

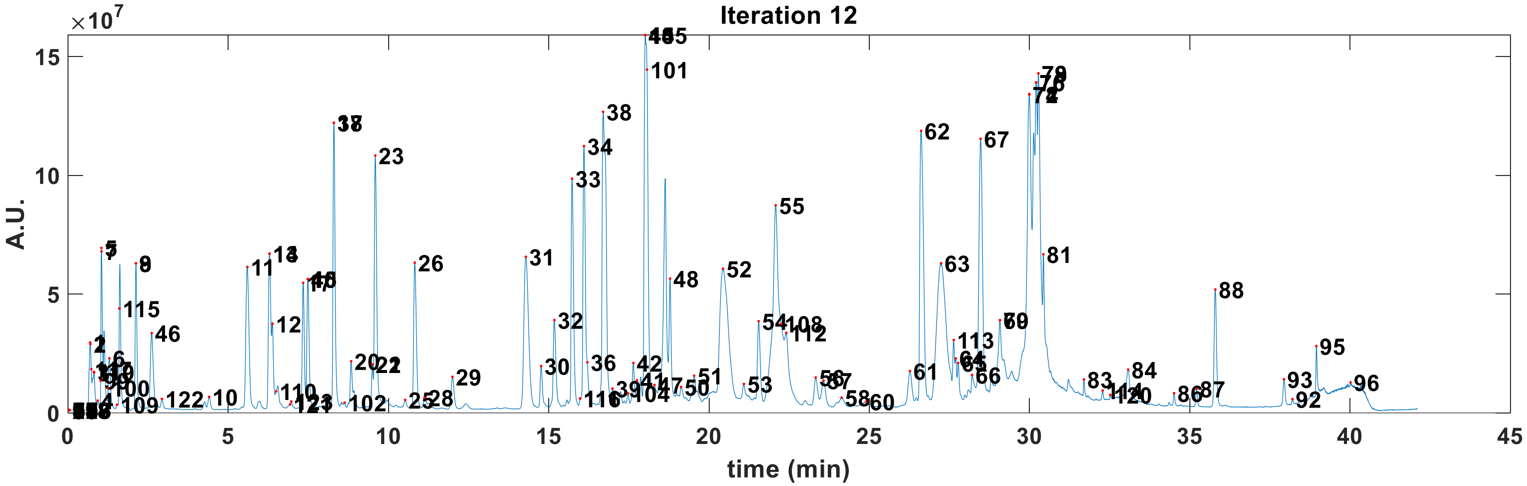

Figure S-12. LC-MS TIC chromatogram of iteration 12.

S-3 Retention modeling constraints

For retention modeling the parameters were constrained. For the LSS model the constraints were [-2,40] and [0,50] for $\ln k_0$ and S, respectively. For the quadratic model the constraints were [-100,20], [0,50] and [0,50] for $\ln k_0$, S, and Q, respectively.

The algorithm would progress with further iterations using the interior-point algorithm until the successive change in the sum of squared errors (SSE) was below 10^{-6}. For each analyte, the best fit from the 20 loops was transferred to the next phase for use in optimization.

S-4 Overview of gradient program

Figure S-13 below shows the schematic of the generalized gradient employed for the retention modeling strategy.

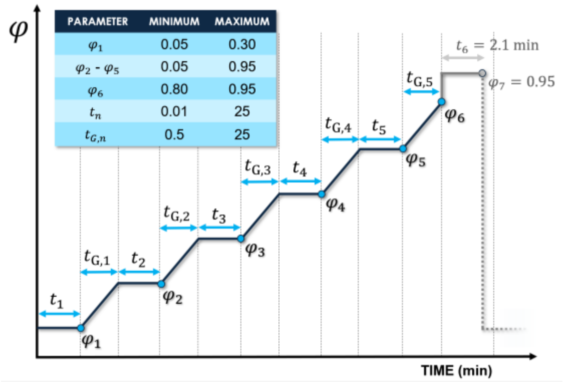

Figure S-13. Schematic of generalized segmented gradient employed for retention modeling. The table indicates the minimum and maximum bounds allowed for each parameter.

As φ_{init} was allowed to vary between 0.05 and 0.30, and φ_2, φ_3, φ_4 and φ_5 were all allowed to vary between 0.05 and 0.95, whereas φ_6 was varied between 0.80 and 0.95. All t_n were allowed to vary between 0.01 and 25 min, and all $t_{G,n}$ between 0.5 and 25 min. Afterwards, the method concluded and φ was set to φ_1 (0.02). There was no controlled re-equilibration, but due to the processing time of the algorithm, the column would receive at least 4 column volumes of eluent A prior to the start of the next iteration.

A minimum φ of 0.05 or 0.02 is common in RPLC and is purely based on the recommendations by the column manufacturer. This is done so the column stationary phase will stay in good condition. φ_{init} was maximized to 0.3 to minimize the search space of the start conditions. This is all done to improve the calculation speed of the algorithm to converge to an optimal gradient program faster (i.e. it saves minutes of computational time). These bounds can be omitted without any problem, but RPLC separations often start with a relatively low modifier fraction. Similarly, the high φ ranges were also pre-selected to ensure that all analytes will elute from the column. Again, this was done to allow the algorithm to be universally applied.

S-5 Predicted score function

This section reflects a graphical exploration of various values for Equation 3 in the main article.

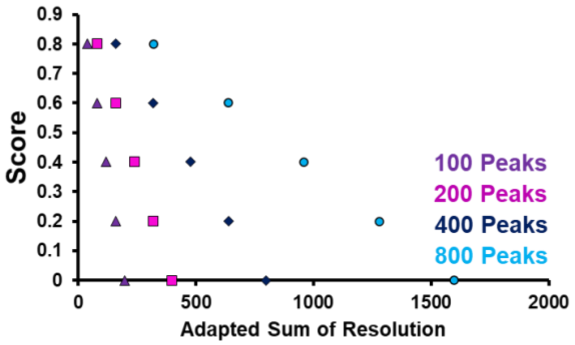

Figure S-14. Theoretical $\boldsymbol{O_{Rs}}$ values for 100 (purple triangles), 200 (pink squares), 400 (dark blue diamonds) or 800 peaks (light blue circles) as a function of adapted sum of resolution.

S-6 Prediction errors in retention time and peak width

The graphs in this section display reconstructions of predicted chromatograms. Note that in these graphs the peak intensity was normalized. The graphs do provide a useful insight in retention times and peak widths. The blue stars depict the magnitude of the prediction error in retention time, whereas the red starts depict this for the peak width (sigma). The second y-axis provides numerical scale to both of these prediction errors. The error in peak width appears always to be below 1%. A systematic negative bias becomes apparent. The error in retention time is, however, rather significant in MDI 4 and MDI 5 for the less retained analytes. This is most likely due to the fact that these predictions are based on peak data which was heavily convoluted in the scouting MDI (i.e. all co-eluting early).

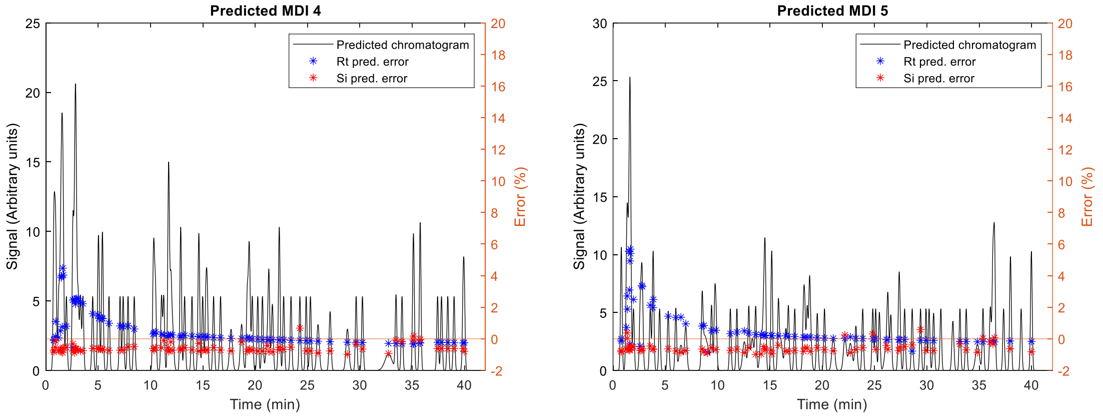

Figure S-15. Reconstructions of predicted chromatograms for MDI 4 (left) and 5 (right), with prediction errors indicated by the blue (retention time) and red (peak width, sigma) stars.

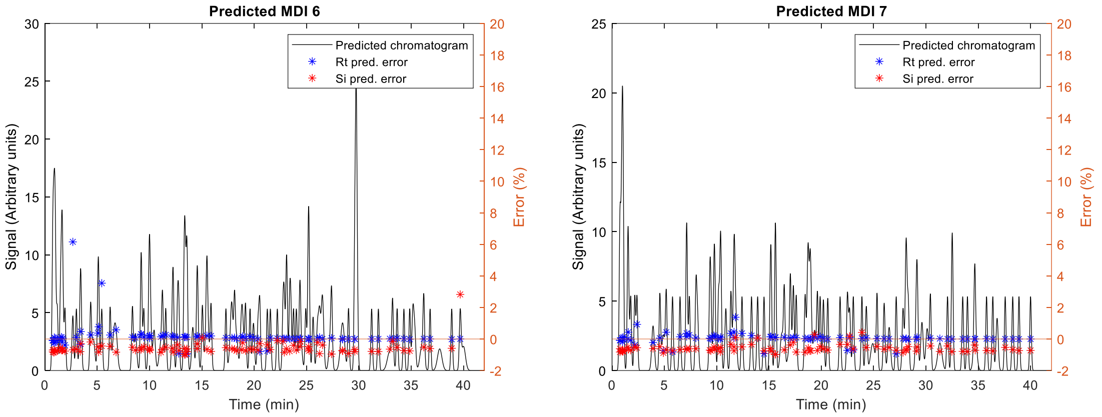

Figure S-16. Reconstructions of predicted chromatograms for MDI 6 (left) and 7 (right), with prediction errors indicated by the blue (retention time) and red (peak width, sigma) stars.

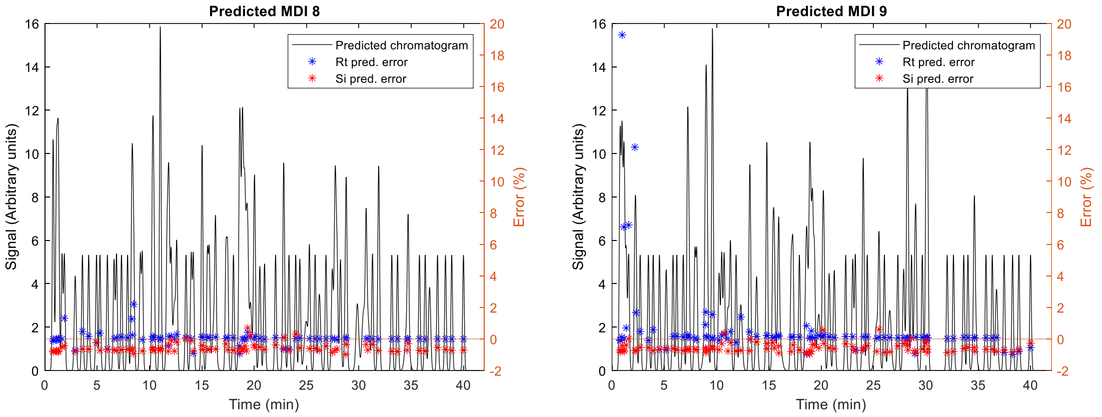

Figure S-17. Reconstructions of predicted chromatograms for MDI 8 (left) and 9 (right), with prediction errors indicated by the blue (retention time) and red (peak width, sigma) stars.

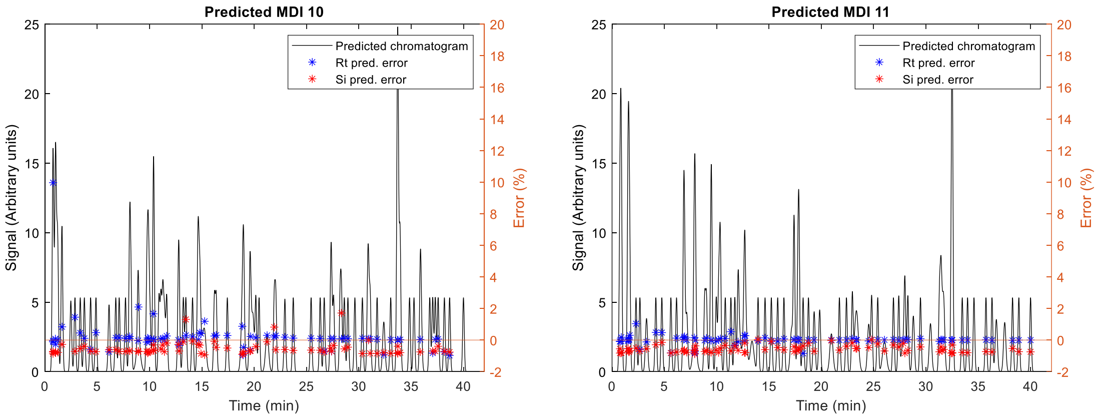

Figure S-18 Reconstructions of predicted chromatograms for MDI 10 (left) and 11 (right), with prediction errors indicated by the blue (retention time) and red (peak width, sigma) stars.

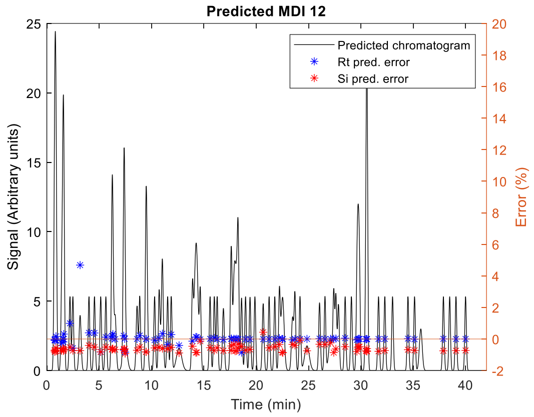

Figure S-19. Reconstructions of predicted chromatograms for MDI 12, with prediction errors indicated by the blue (retention time) and red (peak width, sigma) stars.

S-7 Minimum peak width

Convoluted peaks are detected with a too low peak width if no deconvolution is performed. To ensure a good separation without the addition of deconvolution, which increases the computational time significantly, we implemented a minimal peak width at the base of 0.3 min. Unfortunately, this impaired the algorithm in its capabilities to predict the true width which was often much smaller. Future iterations of this work will have this fixed.

Table S-2. Experimentally and predicted base peak width and the difference between them.

Experimental	Predicted	Difference
**0.055329563**	0.3	-0.244670437
**0.080540428**	0.3	-0.219459572
**0.131686492**	0.3	-0.168313508
**0.055347306**	0.3	-0.244652694
**0.07989361**	0.3	-0.22010639
**0.108383365**	0.3	-0.191616635
**0.075346376**	0.3	-0.224653624
**0.094760806**	0.3	-0.205239194
**0.094760806**	0.3	-0.205239194
**0.143046288**	0.3	-0.156953712
**0.177474133**	0.3	-0.122525867
**0.098836437**	0.3	-0.201163563
**0.145752212**	0.3	-0.154247788
**0.145752212**	0.3	-0.154247788
**0.112509272**	0.3	-0.187490728
**0.112509272**	0.3	-0.187490728
**0.111749572**	0.3	-0.188250428
**0.113909614**	0.3	-0.186090386
**0.10665764**	0.3	-0.19334236
**0.054881426**	0.3	-0.245118574
**0.054881426**	0.3	-0.245118574
**0.129903805**	0.3	-0.170096195

**0.146704672**	0.3	-0.153295328
**0.14434944**	0.3	-0.15565056
**0.162025014**	0.3	-0.137974986
**0.117912211**	0.3	-0.182087789
**0.258591119**	0.3	-0.041408881
**0.140630313**	0.3	-0.159369687
**0.133579197**	0.3	-0.166420803
**0.132284954**	0.3	-0.167715046
**0.09193372**	0.356574333	-0.264640613
**0.081788405**	0.3	-0.218211595
**0.112509272**	0.979480581	-0.866971309
**0.073582352**	0.31811737	-0.244535019
**0.09140695**	0.328458013	-0.237051063
**0.213704038**	0.311525085	-0.097821046
**0.213704038**	0.321161141	-0.107457102
**0.133475394**	0.312101044	-0.178625651
**0.161437421**	0.305689085	-0.144251663
**0.076621151**	0.315060049	-0.238438898
**0.085821779**	0.3	-0.214178221
**0.102612927**	0.302046465	-0.199433538
**0.473673632**	0.331574558	0.142099075
**0.132012823**	0.3	-0.167987177
**0.15268832**	0.3	-0.14731168
**0.275108516**	0.417960911	-0.142852396
**0.193806059**	0.3	-0.106193941
**0.179671431**	0.3	-0.120328569
**0.272293253**	0.3	-0.027706747
**0.225748714**	0.895939492	-0.670190778
**0.196663437**	0.3	-0.103336563
**0.218203923**	0.326917522	-0.108713599
**0.52181171**	0.691760543	-0.169948833
**0.041357474**	0.3	-0.258642526
**0.06260639**	0.3	-0.23739361
**0.111839473**	0.3	-0.188160527
**0.143270634**	0.3	-0.156729366
**0.152707758**	0.3	-0.147292242
**0.152707758**	0.3	-0.147292242
**0.152707758**	0.30632678	-0.153619022
**0.074317754**	0.3	-0.225682246
**0.074317754**	0.3	-0.225682246
**0.116828546**	0.3	-0.183171454
**0.063127975**	0.3	-0.236872025
**0.065910842**	0.3	-0.234089158
**0.084244066**	0.3	-0.215755934

**0.095965149**	0.3	-0.204034851
**0.075263734**	0.3	-0.224736266
**0.077790667**	0.3	-0.222209333
**0.082209561**	0.3	-0.217790439
**0.068033191**	0.3	-0.231966809
**0.060663406**	0.7926179	-0.731954494
**0.105203645**	0.402103053	-0.296899408
**0.098066926**	0.344231072	-0.246164146
**0.213704038**	0.383380166	-0.169676127
**0.095574278**	0.58868575	-0.493111472
**0.121028402**	0.3	-0.178971598
**0.133529494**	0.40725904	-0.273729546
**0.095430949**	0.652434027	-0.557003079
**0.07720105**	0.596413763	-0.519212714
**0.081432043**	0.3	-0.218567957
**0.072261249**	0.3	-0.227738751
**0.095430949**	0.673174991	-0.577744042
**0.087439613**	0.3	-0.212560387

S-8 In-depth study of retention modeling using UV-vis data and the quadratic model

We also investigated whether the number of retention data (i.e. the number of previous MDI) significantly affected the model. We employed the quadratic model (Equation S-1)[4]

$$\ln k = \ln k_0 + S\varphi + Q\varphi^2$$

(S-1)

Measurements were conducted using a mixture of 15 dye standards: Rhodamine B (79754-25MG), Crystal Violet (46364-250MG), Brilliant Green (78364-25MG), Metanil Yellow (44426-100MG), Martius Yellow (377767-25G), Patent Blue V calcium salt (74748-25MG), Congo Red (75768-25MG), Victorya Blue B (94890-25G), Naphthol Yellow S (49547-25MG), Ponceau Xylidine (22308-25MG), Ponceau 6R (96365-25MG), Amaranth (87612-25MG), Acid Red 33 (59573-25MG) and Indigo Carmine (73436-25MG), all of which were obtained from Sigma-Aldrich, and Crocein Orange G (22800-25MG) which was obtained from Chem-Impex International (Wood Dale, IL, United States).

The results are shown in Figures S-20B-D. With adequate peak tracking the already solid models improve with each MDI (Figure S-20B).

However, the data in Figure S-20 were obtained in combination with an investigation of the feasibility of using UV-vis data for peak tracking. For this effort a UV-vis peak tracking algorithm was developed[5]. Figures S-20C and S-20D show the initial description of retention as a function of φ for some analytes was poor and only improved to acceptable limits once data was available for at least 10 MDI (Figure S-20C, Figure S-20D). Poor descriptions of retention were mainly observed for overlapping (i.e., not fully separated) analytes that were difficult to track, as is also shown by the peak-tracking tables (Supporting Information section S-9).

Figure S-20E displays the scores in a bubble plot as a function of iteration for this study (Quadratic model, dark blue). The size of the bubbles indicates the relative length of the gradient time. This explains why with the first five MDI – the scouting gradients for the quadratic model with increasingly longer gradient lengths (Figure S-20A) - the bubbles become increasingly larger. Usually, longer (or shallower) gradients yield better separation, which is also reflected by the first five MDI. Optimization algorithms will therefore often prefer such gradients when the number of gradient segments is limited. However, after MDI 6, once the algorithm was forced to use multi-segment gradients (Figure S-20F), the algorithm exclusively proposed relatively short gradients as also indicated by the small bubbles. Figure 4D also showcases an example of how an exit function – a function that decides whether the automated workflow should stop – can be designed. The log fit through the bubbles (dashed, dark blue line) flattens towards the higher number of MDI. This indicates that with every additional MDI less improvement is obtained by continuing the workflow. Using the first derivative of this function (*i.e.*, the slope of the log function) a threshold can be defined. Once the slope is below this threshold, the automation algorithm can exit the iteration loop.

Finally, it can be seen in Figure S-20E that using the LSS model for the same sample the optimization curve flattens more rapidly. Due to the lack of degrees of freedom, the algorithm is incapable to further fine-tune separation conditions to achieve a better O_{perf}.

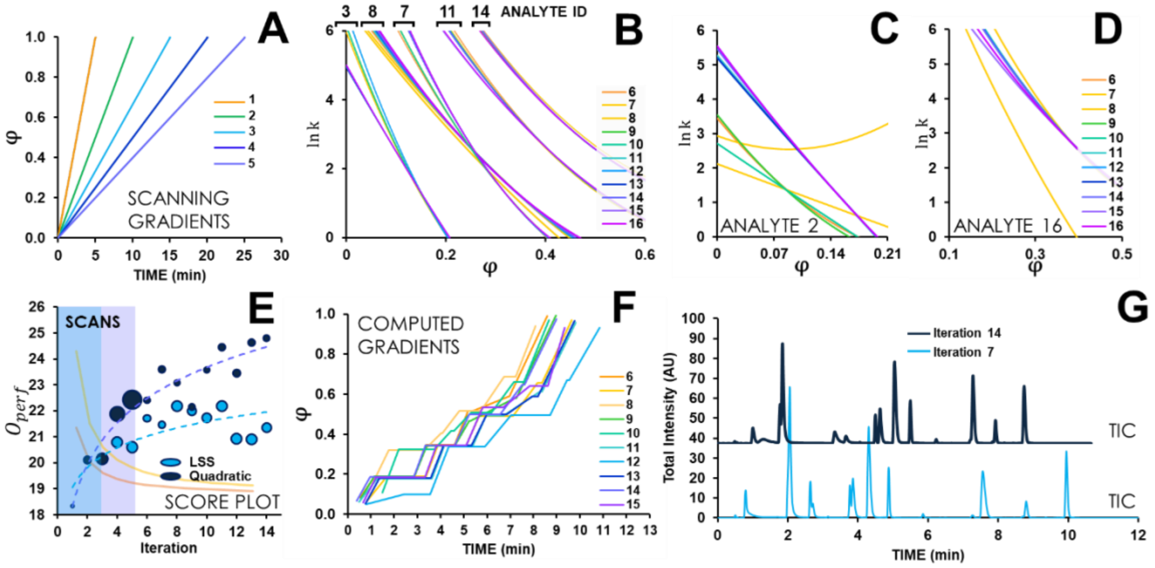

Figure S-20. A) Scouting gradients used in MDIs 1-5. B) Examples of retention plots using the quadratic model for selected compounds and their improvement with every MDI as more data becomes available. C-D) Example of poor regression due to a lack of data points (or poor quality) for analytes 2 (C) and 16 (D), and their improvement with new MDI. E) Bubble plot of scores for the experiment conducted using the quadratic (dark blue) and linear solvent strength (LSS) (light blue) model for retention modeling. Dashed line indicates fitted log function. Size of bubble indicates relative length of method in time. The yellow and orange line plotted on a hidden axis represent the derivatives of the log function (Quadratic and LSS respectively) which indicates how much the score still improves. F) Computed gradients for iterations 6-15. G) Total-intensity chromatograms (TIC) of MDI 7 (light blue) and 14 (dark blue). In this study, we gave the algorithm a budget of 15 MDI to study the performance, but in practice an exit function can be defined (See Figure S-20D). Data measured on System A.

S-9 UV-Vis peak tracking data

The tables below give an overview of the obtained detection and tracking results using the LSS (Table S-2) and Quadratic (Table S-3) models.

Table S-3. Detection and tracking for the experiments using the LSS model. Retention time in min is given per peak and measurement. N.D. = not detected.

# Peak\run	1	2	3	4	5	6	7	8	9	10	11	12	13	14	15
1	1.39	1.74	2.04	0.51	0.70	0.76	0.79	0.74	0.61	0.56	0.76	0.60	0.59	0.60	0.49
2	1.82	2.53	N.D.	1.35	N.D.	1.76	2.00	1.91	1.70	1.65	2.07	1.97	1.87	1.97	1.09
3	1.88	2.59	3.18	1.47	N.D.	1.82	2.05	1.97	1.77	1.76	2.13	2.05	1.94	2.04	1.22
4	2.23	3.33	4.32	6.36	5.76	2.41	2.64	3.50	3.53	4.02	4.78	4.67	4.03	4.13	4.50
5	2.29	3.43	4.45	7.27	6.11	2.48	2.72	3.86	3.92	4.44	5.38	5.14	4.45	4.54	5.14
6	2.82	4.45	N.D.	11.02	10.91	3.53	3.77	6.07	6.10	6.56	6.24	N.D.	N.D.	6.63	8.93
7	2.91	4.51	N.D.	N.D.	N.D.	3.61	3.85	6.01	6.04	6.51	6.32	N.D.	N.D.	6.58	8.10
8	3.09	4.77	6.24	10.52	10.05	4.05	4.30	6.22	6.25	6.70	6.55	6.91	6.10	6.78	8.28
9	3.17	5.05	6.76	11.59	11.58	4.63	4.88	6.86	6.88	7.32	7.11	7.51	6.69	7.38	9.58
10	3.68	5.87	7.88	12.29	12.30	5.74	5.86	8.71	8.46	8.90	9.35	9.60	8.87	8.94	10.36
11	4.13	6.62	8.92	13.24	13.31	7.62	7.54	12.01	11.20	11.61	12.98	13.03	12.40	11.64	11.56
12	4.46	7.11	9.54	13.82	14.00	N.D.	N.D.	N.D.	11.85	12.25	N.D.	13.67	13.02	12.26	12.08
13	4.54	7.19	9.64	13.90	14.09	9.17	8.80	13.93	11.92	12.31	13.73	13.74	13.09	12.33	12.16
14	5.13	8.07	10.83	14.72	15.10	10.30	9.94	14.78	12.76	13.14	14.74	14.69	14.17	13.16	12.94
15	N.D.	1.80	2.14	0.67	0.79	N.D.	N.D.	N.D.	N.D.	N.D.	N.D.	0.79	N.D.	0.79	N.D.
16	N.D.	6.56	8.81	13.15	13.19	7.28	7.26	11.21	10.62	11.03	N.D.	N.D.	N.D.	11.07	11.44
17	N.D.	N.D.	3.15	N.D.	N.D.	N.D.	N.D.	N.D.	N.D.	N.D.	N.D.	N.D.	N.D.	N.D.	N.D.
18	N.D.	N.D.	4.39	N.D.	N.D.	N.D.	N.D.	N.D.	N.D.	N.D.	N.D.	N.D.	N.D.	N.D.	N.D.
19	N.D.	N.D.	5.96	N.D.	N.D.	N.D.	N.D.	N.D.	N.D.	N.D.	N.D.	6.72	N.D.	N.D.	N.D.
20	N.D.	N.D.	9.00	13.31	N.D.	N.D.	N.D.	N.D.	N.D.	N.D.	N.D.	N.D.	N.D.	N.D.	N.D.
21	N.D.	N.D.	N.D.	0.55	N.D.	N.D.	N.D.	N.D.	N.D.	N.D.	N.D.	N.D.	N.D.	N.D.	N.D.
22	N.D.	N.D.	N.D.	N.D.	0.49	N.D.	N.D.	N.D.	0.49	0.48	N.D.	0.49	0.49	0.49	N.D.
23	N.D.	N.D.	N.D.	N.D.	2.95	N.D.	N.D.	N.D.	N.D.	N.D.	N.D.	N.D.	N.D.	N.D.	N.D.
24	N.D.	N.D.	N.D.	N.D.	10.00	N.D.	N.D.	N.D.	N.D.	N.D.	N.D.	N.D.	N.D.	N.D.	N.D.
25	N.D.	N.D.	N.D.	N.D.	13.38	N.D.	N.D.	N.D.	N.D.	N.D.	N.D.	N.D.	N.D.	N.D.	N.D.
26	N.D.	N.D.	N.D.	N.D.	N.D.	0.50	0.50	N.D.	N.D.	N.D.	0.49	N.D.	N.D.	N.D.	N.D.
27	N.D.	N.D.	N.D.	N.D.	N.D.	0.82	0.85	N.D.	N.D.	N.D.	0.81	N.D.	N.D.	N.D.	0.55
28	N.D.	N.D.	N.D.	N.D.	N.D.	N.D.	N.D.	0.49	N.D.	N.D.	N.D.	N.D.	N.D.	N.D.	N.D.
29	N.D.	N.D.	N.D.	N.D.	N.D.	N.D.	N.D.	0.80	N.D.	N.D.	N.D.	N.D.	N.D.	N.D.	N.D.
30	N.D.	N.D.	N.D.	N.D.	N.D.	N.D.	N.D.	N.D.	N.D.	N.D.	N.D.	N.D.	5.89	N.D.	N.D.

Table S-4. Detection and tracking for the experiments using the quadratic model. Retention time in min is given per peak and measurement. N.D. = not detected.

# Peak\run	1	2	3	4	5	6	7	8	9	10	11	12	13	14	15
1	1.38	1.74	2.04	2.29	2.51	0.75	1.01	0.57	0.80	0.61	1.13	1.45	1.30	1.04	1.20
2	1.82	2.53	N.D.	N.D.	N.D.	1.92	2.39	1.72	1.94	2.17	2.12	N.D.	2.26	1.85	2.12
3	1.88	2.59	N.D.	N.D.	N.D.	1.98	2.45	1.83	1.99	2.38	2.18	4.11	2.32	1.92	2.18
4	2.23	3.34	4.40	5.36	6.28	3.37	3.87	2.41	3.39	2.89	4.09	5.01	4.01	3.46	3.78
5	2.29	3.43	4.46	5.43	6.35	3.72	4.22	2.47	3.74	2.94	4.54	5.06	4.43	3.80	4.18
6	2.83	4.46	N.D.	7.41	8.78	4.69	5.22	3.51	4.76	3.90	5.53	5.84	5.45	4.68	5.18
7	2.91	4.52	N.D.	7.33	8.73	4.77	5.29	3.61	4.83	4.01	5.60	5.97	5.52	4.82	5.25
8	3.09	4.76	6.24	7.58	8.85	5.09	5.62	4.09	5.16	4.49	5.92	6.38	5.83	5.25	5.58
9	3.17	5.05	6.77	8.40	9.95	5.63	6.18	4.72	5.75	5.09	6.48	7.04	6.41	5.72	6.16
10	3.68	5.87	7.88	9.78	11.58	6.29	6.94	5.61	6.57	6.04	7.22	7.96	7.16	6.49	6.80
11	4.12	6.61	8.91	11.09	13.18	7.06	8.07	6.55	7.45	7.05	8.16	9.08	8.13	7.58	7.60
12	4.45	7.10	9.54	11.84	14.05	7.65	8.67	7.12	8.06	7.63	8.77	9.87	8.75	8.17	8.18
13	4.53	7.18	9.62	11.94	14.15	7.74	8.74	7.19	8.14	7.71	8.85	9.95	8.83	8.25	8.27
14	5.11	8.05	10.82	13.44	15.97	8.57	9.64	8.10	8.96	8.65	9.81	10.84	9.76	9.10	9.38
15	N.D.	1.80	2.14	2.44	2.71	N.D.	1.22	0.68	N.D.	0.79	N.D.	N.D.	N.D.	N.D.	N.D.
16	N.D.	6.55	8.80	10.92	12.93	7.00	7.96	6.46	7.38	6.94	8.08	8.94	8.05	7.51	7.53
17	N.D.	N.D.	3.16	N.D.	N.D.	N.D.	N.D.	N.D.	N.D.	N.D.	N.D.	4.31	N.D.	N.D.	N.D.
18	N.D.	N.D.	3.18	3.71	4.20	N.D.	N.D.	N.D.	N.D.	2.25	N.D.	N.D.	N.D.	N.D.	N.D.
19	N.D.	N.D.	4.33	5.26	N.D.	N.D.	N.D.	N.D.	N.D.	N.D.	N.D.	N.D.	N.D.	N.D.	N.D.
20	N.D.	N.D.	5.97	N.D.	N.D.	N.D.	N.D.	N.D.	N.D.	N.D.	N.D.	N.D.	N.D.	N.D.	N.D.
21	N.D.	N.D.	N.D.	N.D.	6.16	N.D.	N.D.	N.D.	N.D.	N.D.	N.D.	N.D.	N.D.	N.D.	N.D.
22	N.D.	N.D.	N.D.	N.D.	8.62	N.D.	N.D.	N.D.	N.D.	N.D.	N.D.	N.D.	N.D.	N.D.	N.D.
23	N.D.	N.D.	N.D.	N.D.	N.D.	0.81	N.D.	N.D.	0.88	N.D.	N.D.	N.D.	N.D.	N.D.	N.D.
24	N.D.	N.D.	N.D.	N.D.	N.D.	N.D.	2.42	N.D.	N.D.	N.D.	N.D.	N.D.	N.D.	N.D.	N.D.
25	1.38	1.74	2.04	2.29	2.51	0.75	1.01	0.57	0.80	0.61	1.13	1.45	1.30	1.04	1.20
26	1.82	2.53	N.D.	N.D.	N.D.	1.92	2.39	1.72	1.94	2.17	2.12	N.D.	2.26	1.85	2.12
27	1.88	2.59	N.D.	N.D.	N.D.	1.98	2.45	1.83	1.99	2.38	2.18	4.11	2.32	1.92	2.18
28	2.23	3.34	4.40	5.36	6.28	3.37	3.87	2.41	3.39	2.89	4.09	5.01	4.01	3.46	3.78
29	2.29	3.43	4.46	5.43	6.35	3.72	4.22	2.47	3.74	2.94	4.54	5.06	4.43	3.80	4.18
30	2.83	4.46	N.D.	7.41	8.78	4.69	5.22	3.51	4.76	3.90	5.53	5.84	5.45	4.68	5.18

S-10 Gradient design of Bayesian Optimization

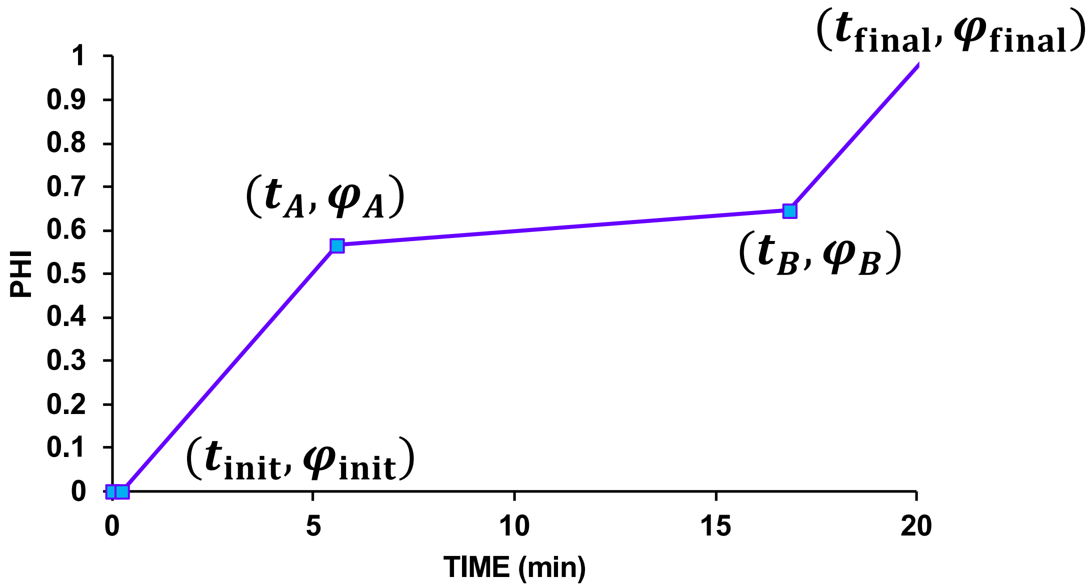

Figure S-21. Schematic of gradient design for Bayesian optimization experiments.

S-11 Gradient deformation

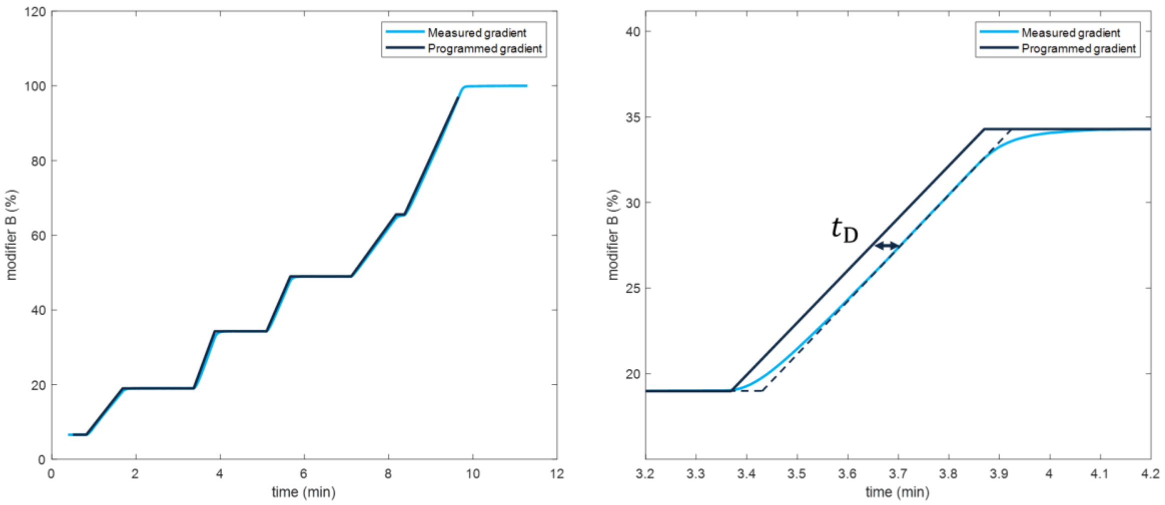

Figure S-22. Left: dark blue is the programmed gradient whereas the light blue is the measured and calibrated C4D-detected modifier composition. Right; zoomed in section showing the deviation from the programmed gradient. Besides the dwell time (t_D) there are more gradient deformations at the beginning and end of the section.

S-12 Algorithm

The *.zip package contains all used code to construct the master algorithm in the various programming languages. The master code is run within Python and configuration for the LC system requires the Automation package freely provided by Agilent. We would like to emphasize that this is a work-in-progress prototype released for transparency and scientific proliferation, and that future (user-friendly) versions will be released on https://www.cast-amsterdam.org/. To use this prototype significant tailoring of the individual scripts will be required.

The UV-vis toolbox, used in this study for the data in Sections S-8 and S-9, by Denice van Herwerden can be downloaded elsewhere[5].

Further design considerations

Analytical instruments comprise sophisticated hardware with safety mechanisms in place to avoid improper use. This is pivotal for industrial environments. For this reason, we opted to interface with existing instrument control software (ICS). However, ICS is often the product of several stages of development and continuously adapted to new technology. To prevent the algorithm from requiring adaptation to specific ICS and its procedures, the algorithm was designed to program and activate the ICS after which a listener function was activated, while the algorithm would remain dormant. Once the LC experiment was finished, the ICS was programmed to create a signal for the listener function to reactivate the algorithm.

Next to design considerations such as being independent and interpretive, flexibility towards metrics published in the literature is also an important consideration point. To this end, the algorithm should be modular. This was achieved by defining a chain of independent operations with controlled input and output criteria. For example, any background-correction algorithm in the literature can be used if its input is a raw signal, and its output a processed signal. This is less trivial than it appears as some background-correction algorithms require data-specific parameters to be defined. To be interpretive, each metric must include additional subroutines to interpret the signal characteristics and self-determine the parameters, rendering various strategies more challenging.

References

(1) Molenaar, S. R. A.; Dahlseid, T. A.; Leme, G. M.; Stoll, D. R.; Schoenmakers, P. J.; Pirok, B. W. J. Peak-Tracking Algorithm for Use in Comprehensive Two-Dimensional Liquid Chromatography – Application to Monoclonal-Antibody Peptides. *J. Chromatogr. A* **2021**, *1639*, 461922. https://doi.org/10.1016/j.chroma.2021.461922.

(2) Pirok, B. W. J.; den Uijl, M. J.; Moro, G.; Berbers, S. V. J.; Croes, C. J. M.; van Bommel, M. R.; Schoenmakers, P. J. Characterization of Dye Extracts from Historical Cultural-Heritage Objects Using State-of-the-Art Comprehensive Two-Dimensional Liquid Chromatography and Mass Spectrometry with Active Modulation and Optimized Shifting Gradients. *Anal. Chem.* **2019**, *91* (4), 3062–3069. https://doi.org/10.1021/acs.analchem.8b05469.

(3) Pirok, B. W. J.; Molenaar, S. R. A.; Roca, L. S.; Schoenmakers, P. J. Peak-Tracking Algorithm for Use in Automated Interpretive Method-Development Tools in Liquid Chromatography. *Anal. Chem.* **2018**, *90* (23), 14011–14019.

https://doi.org/10.1021/acs.analchem.8b03929.

(4) P.J. Schoenmakers, H.A.H. Billier, R. Tijssen, L. de G. Gradient Selection in Reversed-Phase Liquid Chromatography. *J. Chromatogr. A* **1978**, *149*, 519–537. https://doi.org/10.1016/S0021-9673(00)81008-0.

(5) Van Herwerden, D. UVvisToolBox https://bitbucket.org/Denice_van_Herwerden/uvvistoolbox/src/master/.
